# Effectiveness of mHealth Interventions to Improve Follow-Up and Management Among Solid Organ Transplant Recipients: Systematic Review and Meta-Analysis

**DOI:** 10.2196/69795

**Published:** 2025-12-17

**Authors:** Xiaohong Lin, Haiya Sun, Jiaxin Fang, Zhufeng Han, Zhenshan Ding, Jianding Guo, Lei Dong, Xiangru Li, Hongxia Liu

**Affiliations:** 1 School of Traditional Chinese Medicine Beijing University of Chinese Medicine Beijing China; 2 College of Nursing Jining Medical University Jining China; 3 School of Nursing Beijing University of Chinese Medicine Beijing China; 4 Department of Urology China-Japan Friendship Hospital Beijing China; 5 School of Computing and Artificial Intelligence Southwest Minzu University Chengdu China

**Keywords:** solid organ transplantation, mobile health, follow-up, self-management, systematic review

## Abstract

**Background:**

Effective follow-up and management after organ transplantation are crucial for transplant recipients. Mobile health (mHealth) interventions have emerged as a significant approach for facilitating follow-up and management. However, there is a lack of systematic reviews and meta-analyses of their effectiveness.

**Objective:**

This study aimed to systematically review and synthesize evidence regarding the effectiveness of mHealth interventions in enhancing follow-up and management for transplant recipients.

**Methods:**

This study included both randomized controlled trials (RCTs) and nonrandomized studies of interventions (NRSIs) that compared the effects of mHealth interventions with usual care in transplant recipients by searching PubMed, Web of Science, Scopus, Embase, CINAHL, and CENTRAL from database inception to June 2025. The primary outcomes included self-care ability, medical regimen adherence, self-monitoring, communication and counseling, medication adherence, physical activity, nutrition, all-cause mortality, complications, rehospitalization, and emergency and outpatient department visits. The risk of bias for each study was assessed using version 2 of the Cochrane risk-of-bias tool for RCTs and the Risk of Bias in Nonrandomized Studies of Interventions tool for NRSIs. Data extraction and quality assessment were conducted by 2 reviewers independently. Data synthesis was conducted using Review Manager. Both a meta-analysis and a narrative synthesis were carried out.

**Results:**

A total of 23 studies (n=15, 65% RCTs and n=8, 35% NRSIs) with 2022 transplant recipients were included. Compared to the control group, mHealth interventions significantly improved self-care ability (mean difference 14.49, 95% CI 9.61-19.36; *P*<.001) and reduced rehospitalization (odds ratio [OR] 0.49, 95% CI 0.34-0.71; *P*<.001). The meta-analysis demonstrated no statistically significant difference in mortality rates (OR 0.73, 95% CI 0.39-1.35; *P*=.31), rejection (OR 0.55, 95% CI 0.25-1.19; *P*=.13), or infection (OR 0.33, 95% CI 0.06-1.82; *P*=.20) between the mHealth intervention and control groups. The narrative synthesis indicated that mHealth interventions could effectively promote adherence to medical regimens and medications, facilitate self-monitoring, and improve communication and consultation.

**Conclusions:**

mHealth interventions significantly improved self-care ability and reduced rehospitalization rates among organ transplant recipients. However, these interventions did not demonstrate a significant effect on all-cause mortality or complications. mHealth interventions showed potential benefits for various self-management behaviors in organ transplant recipients, but these findings need to be further verified. Future research should prioritize high-quality studies that investigate the impact of mHealth on physical activity, nutrition, and other patient-centered outcomes.

**Trial Registration:**

International Platform of Registered Systematic Review and Meta-Analysis Protocols INPLASY202480101; https://inplasy.com/inplasy-2024-8-0101/

**International Registered Report Identifier (IRRID):**

RR2-10.37766/inplasy2024.8.0101

## Introduction

### Background

Solid organ transplantation is a life-saving treatment for patients with end-stage organ failure. According to the latest 2022 data from the Global Observatory on Donation and Transplantation, over 150,000 solid organ transplants are performed worldwide each year [1]. Although the short-term survival rate of most solid organ transplant recipients has improved drastically, numerous challenges remain for their long-term survival, such as rejection, infection, complications, and adverse events [2,3]. Transplant recipients require continuous medical management, which includes the lifelong administration of immunosuppressants, consistent and regular follow-up appointments, and the execution of complex self-management tasks [4-6]. Long-term and effective posttransplant follow-up and management are crucial for enhancing both the survival rates and quality of life of organ transplant recipients.

Traditional methods of follow-up and management, including outpatient and telephone follow-ups, have several limitations. Outpatient follow-up is constrained by temporal and geographical factors, necessitating frequent patient travel to and from the hospital, thereby increasing time and financial burdens [7]. Although telephone follow-up is convenient, it suffers from incomplete information records and cannot monitor patients’ physiological indicators in real time. With the burgeoning prevalence of smart devices and mobile apps, mobile health (mHealth) technology has been progressively used in the management of organ transplantation [8]. The World Health Organization has defined mHealth as the “medical and public health practice supported by mobile devices, such as mobile phones, patient monitoring devices, PDAs, and other wireless devices” [9].

With the advantages of device ubiquity, mobility, and portability, mHealth technology enables information transmission and communication between patients and physicians [10]. mHealth interventions offer personalized health monitoring and management services to patients via smart devices and mobile apps [11]. Through continuous real-time monitoring of recipients’ physiological indicators such as blood pressure, blood sugar levels, temperature, and lung capacity, physicians can promptly detect abnormalities, adjust immunosuppressant dosage regimens accordingly, and effectively prevent rejection and other complications. mHealth interventions offer a suite of functionalities that include smart reminders, motivational design elements, and personalized feedback, which have been instrumental in enhancing medication and exercise adherence across diverse populations [12,13].

### Objectives

Numerous studies have explored the application of mHealth interventions in one or multiple aspects of follow-up and management among transplant recipients. However, the results of these studies exhibit significant variability. While some studies have demonstrated a notable impact of mHealth interventions [14-16], others have found no substantial effects in some aspects [17,18]. Furthermore, the extent of sustained use of mHealth technologies varies considerably across different studies. Therefore, while the deployment of mHealth interventions in organ transplantation management holds promise, their effectiveness requires systematic review and further validation.

Two systematic reviews have examined the impact and effectiveness of IT-based interventions on self-management in renal transplant recipients [19,20]. While IT-based interventions encompass a broad spectrum of health-related activities that use various IT tools, mHealth interventions are more narrowly focused. One systematic review evaluated the effectiveness of mHealth-based self-management apps following organ transplantation [21]. However, it primarily focused on 3 aspects of self-management—medication adherence, medical regimen adherence, and remote monitoring—lacking a comprehensive summary of other self-management behaviors, such as physical activity management, or other health outcomes. This study aimed to evaluate the effectiveness of mHealth interventions in improving follow-up and management for solid organ transplant recipients through a systematic review.

## Methods

The protocol of this systematic review and meta-analysis was registered in the International Platform of Registered Systematic Review and Meta-Analysis Protocols (INPLASY202480101). We followed the PRISMA (Preferred Reporting Items for Systematic Reviews and Meta-Analyses) guidelines and checklist [22].

### Search Strategy

We searched PubMed, Web of Science Core Collection, Scopus, Embase, CINAHL, and CENTRAL from database inception to June 2025 to identify all available controlled trials that used mHealth interventions in the follow-up and management of solid organ transplant recipients. Additionally, we examined the reference lists of the included studies and relevant reviews to identify gray literature. The search strategies used a combination of MeSH (Medical Subject Headings) and title and abstract words adapted for each database and were discussed by reviewers with literature search expertise (Multimedia Appendix 1). The key search strings consisted of 3 concepts: “mobile health,” “organ transplantation,” and “follow-up and management.” A comprehensive search strategy was implemented incorporating all possible spellings and synonyms and using Boolean logic for combining terms.

### Selection Criteria

The inclusion criteria were as follows: (1) the participants were recipients who had undergone solid organ transplantation (including lung, kidney, liver, heart, and pancreas transplants); (2) the interventions were mHealth interventions for follow-up and management, covering outpatient visits, self-management, medication adherence, self-monitoring, exercise, and diet, via mobile devices such as phones, tablets, or wearable devices; (3) the comparison was to usual care, which involved routine follow-up management without mHealth technology or only used mobile devices to receive SMS text messages or group messages; (4) the outcomes were various health care metrics such as self-care ability, medical regimen adherence, self-monitoring, communication and counseling, medication adherence, physical activity, nutrition, all-cause mortality, complications, rehospitalization, and emergency and outpatient department visits as the primary outcome and recipients’ retention rate, adoption and use, and satisfaction and acceptance as the secondary outcome; and (5) the study designs were randomized controlled trials (RCTs) and nonrandomized studies of interventions (NRSIs)

The exclusion criteria were as follows: (1) interventions that solely used mobile devices for texting or videoconferencing in the intervention group as these technologies are already widely used; (2) research that was incomplete or lacked available full text; and (3) editorials, reviews, protocols, letters to the editor, commentaries, and books.

### Study Selection

The search results from all databases were imported into EndNote (version 20; Clarivate Analytics) for deduplication and literature management. Duplicate records were removed using both EndNote’s automated tools and subsequent manual checks by researchers to ensure accuracy. Two independent reviewers (X Lin and JF) initially screened the titles and abstracts against the predefined inclusion criteria. Studies deemed potentially eligible were further assessed through full-text review. The 2 reviewers (X Lin and JF) then discussed their selections to reach a consensus, with a third reviewer (X Li) available for arbitration in case of disagreements.

### Data Extraction

Data were extracted using a standardized data extraction form (Multimedia Appendices 2-4 [14-18,23-42]). This form was developed by the review team based on the Cochrane Handbook for Systematic Reviews of Interventions [43] and was piloted on 3 of the included studies. The data included the following domains: study characteristics (author, publication year, country, and study design), participant information (transplantation type, age, and sample size), intervention and control details (type of intervention, mHealth tools and features, intervention description, duration of the interventions, and assessment periods), and outcomes (measurement tools, intervention results, and effects). Two reviewers (X Lin and JF) extracted the data independently and cross-checked them for accuracy. Disagreements were discussed and consulted with a third reviewer (X Li) to reach a consensus.

### Risk of Bias

Two reviewers (X Lin and JF) independently assessed the risk of bias in the selected studies using one of the following instruments based on the study design. For RCTs, version 2 of the Cochrane risk-of-bias tool for randomized trials was used, which examines 5 domains of bias, with each component evaluated as having a low risk of bias, some concerns, or a high risk of bias [44]. For NRSIs, the Risk of Bias in Nonrandomized Studies of Interventions tool recommended by Cochrane was used, which evaluates 7 bias domains, with each component evaluated as having a low risk, moderate risk, serious risk, critical risk, or no information [45]. The overall bias judgment was evaluated according to domain-level judgments. A third reviewer (X Li) was consulted to resolve disagreements.

### Statistical Analysis

We conducted the meta-analyses using Review Manager (version 5.4; The Cochrane Collaboration). We synthesized data by measuring effects for the outcomes of the included studies. For dichotomous variables, odds ratios (ORs) and 95% CIs were used as effect sizes. For continuous variables, the mean difference or standardized mean difference was used to calculate the intervention effect. We assessed heterogeneity between studies using both the chi-square test and *I*^2^ statistic (*I*^2^=0%-100%; values of >50% were considered as substantial statistical heterogeneity). The random-effects model was used when the heterogeneity was significantly high, and the fixed-effects model was used for outcomes with low heterogeneity. As the number of studies in each comparison was less than 10, funnel plot asymmetry was not tested. Due to the heterogeneity in the nature of interventions and the diverse outcomes of follow-up and management, we also conducted a narrative review of the findings.

## Results

### Study Selection

**Figure 1:**
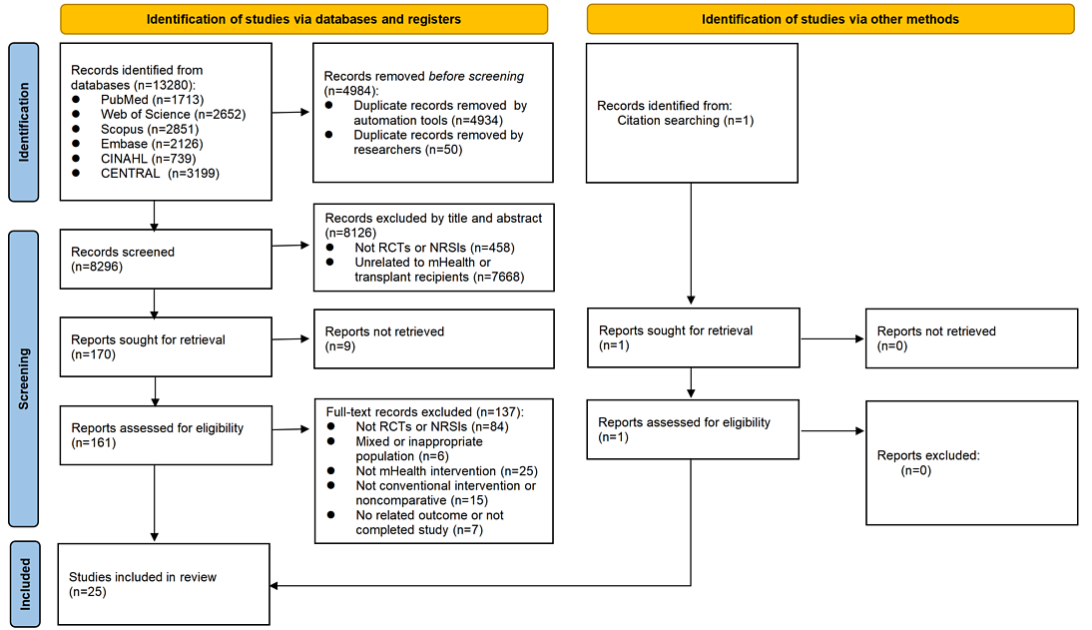
PRISMA (Preferred Reporting Items for Systematic Reviews and Meta-Analyses) flow diagram for study selection. mHealth: mobile health; NRSI: nonrandomized study of interventions; RCT: randomized controlled trial.

A total of 13,280 records were retrieved from the databases. After removing duplicates, of these 13,280 records, 8296 (62.47%) potentially eligible articles were screened based on titles and abstracts. Of these 8296 articles, 161 (1.94%) underwent full-text review, of which 24 (14.9%) met the inclusion criteria. We additionally tracked relevant citations in the included studies. Finally, 25 articles met the inclusion criteria for the systematic review (n=17, 68% RCTs and n=8, 32% NRSIs). Figure 1 shows the PRISMA flow diagram detailing the selection process.

### Study Characteristics

The characteristics of the 25 articles are shown in Multimedia Appendix 2. Of the 25 articles, 2 [23,24] originated from the same study, and another 2 [25,26] similarly originated from the same study. Consequently, these 4 articles were combined into 2 studies. Of the resulting 23 studies, 12 were from the United States; 4 were from China; and 2 were from South Korea; there were 1 each from Spain, Sweden, the United Kingdom, Germany, and Canada. A total of 2022 organ transplant recipients were included in the studies, with 1020 recipients allocated to the mHealth intervention group and 1002 to the control usual care group. Nine studies focused on kidney transplant recipients, 6 included lung transplant recipients, 3 recruited liver transplant recipients, 2 involved heart transplant recipients, and 3 encompassed multiple or combined organ transplant recipients.

### Risk of Bias

The results of the risk-of-bias assessment are summarized in Multimedia Appendix 5. Of the 15 RCTs assessed using version 2 of the Cochrane risk-of-bias tool, 2 showed a low risk of bias, 6 exhibited some concerns about the risk of bias, and the remaining 7 showed a high risk of bias. Nine (60%) of the studies [17,18,23,31-35,41] were judged to have some concerns or high risk in the randomization process because they did not describe allocation concealment, lacked detailed information about randomization, or reported baseline differences. Blinding of participants was not possible in any of the studies due to the nature of the mHealth intervention. Three studies reported missing data from baseline to the end point [18,27,28], and the reasons for participant dropout in one study [28] were associated with the intervention. One study [27] was judged as “high risk” for the measurement of the outcome because the primary outcome measures were assessed using mHealth apps; however, the use of these apps was notably low in the intervention group [32]. In addition, 2 studies did not report all the outcomes as described in the Methods section, resulting in judgment of having some concerns or high risk regarding selection of the reported results [30,32].

Regarding the 8 NRSIs assessed using the Risk of Bias in Nonrandomized Studies of Interventions, all studies were at serious risk of bias. The risk of bias in NRSIs was mainly due to grouping by participants’ preferences or the COVID-19 outbreak [16,36,38-40]. Outcome measures in 3 studies were subjective scores reported by patients, and no blinding method was reported [15,40,42]. One study did not report the duration of the intervention [42]. The outcome data presented in one study did not align with the descriptions provided in the Methods section, and a comparative analysis of baseline data between the 2 groups was absent [15]. More detailed information on the risk-of-bias assessment is provided in Multimedia Appendix 5 [14-18,23-42].

### Intervention

The types of interventions in these studies can be classified into four categories: (1) self-care or self-management [14-17,42], (2) adherence to medication or medical regimen [18,23-28,31-33,36,38,40], (3) home self-monitoring [14,17,30,34,35,37], and (4) physical activity and rehabilitation [29,39,41] (Multimedia Appendix 2). These studies used a range of mHealth technologies for the interventions, including (1) health mobile apps deployed on various mobile devices such as smartphones, smartwatches, and tablets; (2) Bluetooth technology for data collection from wearable health monitoring devices to mobile platforms; (3) smart robots equipped with multiple functionalities; and (4) electronic medication trays integrated with mobile devices. The duration of the interventions ranged from 2 weeks to 24 months. Of the 23 studies, 5 (22%) had interventions lasting ≥12 months [17,18,23-26,28] and 2 (9%) did not mention the duration of the interventions [15,42]. Additionally, 5 (22%) of the studies examined long-term outcomes evaluated at 6 months or longer after the end of the intervention [18,32,33,35,37].

### Outcome Measures

#### Self-Care Ability

**Figure 2:**
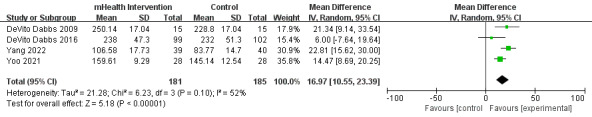
Forest plot of the effects of mobile health (mHealth) interventions on self-care ability. IV: inverse variance.

Five studies reported self-care ability. Of these 5 studies, 2 used the questionnaire on perception of self-care agency [14,17], 1 used the Kidney Transplant Self-Management Scale [42], and 1 used a tool developed specifically for heart transplant recipients [16]. Another study used the Chronic Kidney Disease Self-Management Behavior Scale, demonstrating a positive intervention effect [15]. However, due to a lack of available numerical data, this study was not included in the meta-analysis [15]. Higher scores across these scales indicate that the recipients have higher self-care ability. The meta-analysis demonstrated a significant improvement in self-care ability among recipients in the mHealth intervention group compared with the control group (mean difference 16.97, 95% CI 10.55-23.39; *P<*.001), with high heterogeneity among the 4 studies (*P=*.10; *I*^2^=52%; Figure 2 [14,16,17,42]; Multimedia Appendix 3).

#### Medical Regimen Adherence

Four studies reported medical regimen adherence, 3 of which used the Health Habits Survey [14,17,18]. However, specific data were not reported in 1 of the studies, and the other 2 reported different types of data. The remaining study used the donation after circulatory death liver transplant patient evaluation questionnaire [38]. Meta-analysis was not possible because these studies presented data differently. On the whole, 3 of the studies showed positive intervention effects [14,17,38], and 1 showed no effect [18] (Multimedia Appendix 3).

#### Self-Monitoring

Eight studies dealt with self-monitoring, including monitoring of vital signs, blood sugar, blood oxygen saturation, lung function, and activity levels [14,17,29,30,34,35,37,39].Three studies reported self-monitoring data, of which 2 showed positive intervention effects [14,17] and 1 showed no effect [34]. Due to a failure to report relevant data in 1 of the studies and distinct data types in the other 2, meta-analysis could not be conducted. Self-monitoring measures in these studies were calculated using objectively recorded data from mobile devices rather than subjective self-reports (Multimedia Appendix 3).

#### Communication and Counseling

Four studies reported communication and counseling outcomes using diverse measurement methodologies. Overall, mHealth interventions appeared to have a positive effect on increasing the number of physician-patient communications [14,17,34], as well as helping shorten the length of each follow-up visit [38] and the time interval for active communication after symptom onset [34] (Multimedia Appendix 3).

#### Medication Adherence

Seven studies reported medication adherence. Of these 7 studies, 4 reported a substantial improvement in medication adherence in the intervention groups [24,25,31,33], 2 suggested that there were no statistically significant differences between the mHealth intervention group and the control group (*P*=.89 [27] and *P*=.19 [40]), and 1 only reported adherence in the intervention group [28]. Eight of the studies used immunosuppressive medication blood concentration and its coefficient of variation (intrapatient variability) to evaluate medication compliance [23-28,33,34,36,40]. Only 2 of these studies reported reduced concentration variability in the intervention group [25,26,33], and 6 indicated no significant difference in blood concentration or variability between the intervention and control groups [23,24,27,28,34,36,40]. Additionally, 2 studies focused on adherence to hypertension medications and measured blood pressure before and after the intervention. The findings indicated that systolic blood pressure was significantly reduced in the mHealth intervention group compared to the control group during the intervention period [31]. However, no significant differences in blood pressure were observed between the groups 12 months after the intervention [32] (Multimedia Appendix 3).

#### Physical Activity

Three studies focused on physical activity. One study reported that a tele-coaching intervention improved movement intensity and increased time spent in at least light activity in recipients more than usual care (with a significant difference) while also helping reduce time spent in sedentary activity and increase daily steps (with a nonsignificant difference) [29]. Another study examined alterations in lower-limb strength and function in recipients undergoing center-based rehabilitation and tele-rehabilitation; however, it did not conduct a comparative analysis between the groups [39]. The findings indicated that the tele-rehabilitation group had significant changes in the 6-minute walk test, gait speed, and quadriceps torque before and after intervention, whereas the center-based rehabilitation group demonstrated significant changes solely in the 6-minute walk test [39]. In another study, as the control group did not use wearable trackers, the daily step counts of the mobile intervention group and the control group were not compared [42] (Multimedia Appendix 3).

#### All-Cause Mortality

**Figure 3:**
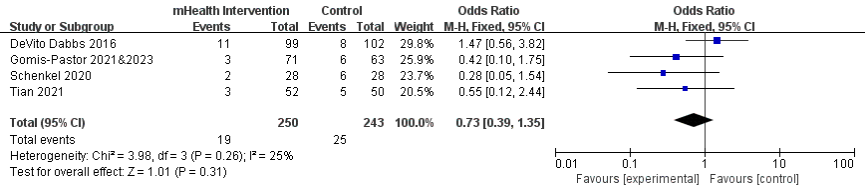
Forest plot of the effects of mobile health (mHealth) interventions on all-cause mortality. M-H: Mantel-Haenszel.

Four studies reported all-cause mortality data [17,23,24,35,37]. The 4 studies included 493 participants and showed low heterogeneity (*P*=.26; *I*^2^=25%). The meta-analysis showed no statistically significant difference in mortality rates between the mHealth intervention and control groups (OR 0.73, 95% CI 0.39-1.35; *P*=.31; Figure 3 [17,23,24,35,37]; Multimedia Appendix 3).

#### Complications

**Figure 4:**
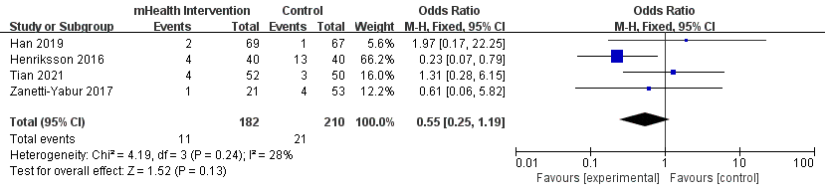
Forest plot of the effects of mobile health (mHealth) interventions on rejection. M-H: Mantel-Haenszel.

**Figure 5:**
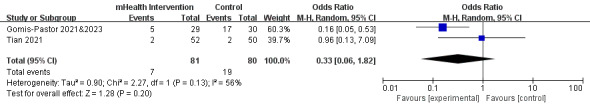
Forest plot of the effects of mobile health (mHealth) interventions on infection. M-H: Mantel-Haenszel.

Six studies reported various complications, including rejection and infection. Four of these studies reported rejection, and the heterogeneity among the studies was low (*P*=.24; *I*^2^=28%) [27,28,35,40]. The meta-analysis showed a nonsignificant trend favoring the intervention group (OR 0.55, 95% CI 0.25-1.19; *P*=.13; Figure 4 [27,28,35,40]; Multimedia Appendix 3). Three of the studies reported infection [23-26,35], but one of them could not be included in the meta-analysis because it measured the rate of infection per patient per year. The remaining 2 of these studies [23,24,35] showed significantly high heterogeneity (*P*=.13; *I*^2^=56%) and no significant between-group difference (OR 0.33, 95% CI 0.06-1.82; *P*=.20; Figure 5 [23,24,35]; Multimedia Appendix 3). Two of the studies reported complications beyond rejection and infection, and the incidence of these complications did not exhibit a statistically significant difference between the 2 groups [24,35] (Multimedia Appendix 3).

#### Rehospitalization

**Figure 6:**
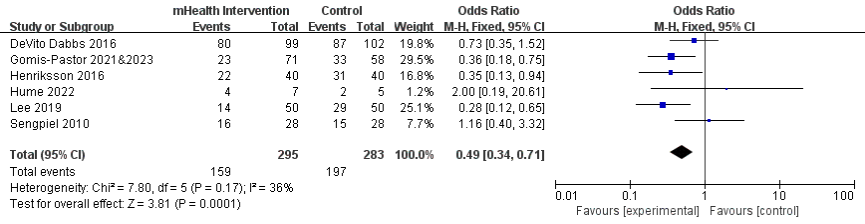
Forest plot of the effects of mobile health (mHealth) interventions on rehospitalization. M-H: Mantel-Haenszel.

Nine studies reported rehospitalization data. Of these 9 studies, 6 reported rehospitalization rates and were included in the meta-analysis. The 6 studies included 578 participants and exhibited low heterogeneity (*P*=.17; *I*^2^=36%) [17,23,24,28-30,34]. The meta-analysis showed that the rehospitalization rate was significantly lower in the mHealth intervention group than in the control group (OR 0.49, 95% CI 0.34-0.71; *P*<.001; Figure 6 [17,23,24,28-30,34]). Results from the other 3 studies that reported rehospitalization rates in other ways were consistent with the conclusions of the meta-analysis [25,26,35,37] (Multimedia Appendix 3).

#### Emergency and Outpatient Department Visits

Two studies reported emergency department visit data, one showing a significantly lower visit rate in the intervention group than in the control group and the other showing no significant difference between the 2 groups [23,24,34]. Four studies reported outpatient visits, all of which showed no significant difference in the rate of outpatient visits between the intervention and control groups [23,24,28,34,37] (Multimedia Appendix 3).

#### Other Outcomes

Retention rates were reported in 8 studies [25-29,31,33,35,41], ranging from 73.2% to 99% in the mHealth intervention group and 80.6% to 100% in the control group. In most studies (5/8), retention rates in the intervention group were slightly lower than those in the control group (Multimedia Appendix 4). Eight studies mentioned the adoption and use rate of mHealth interventions [16-18,23,24,27,30,31,37], 4 of which involved long-term use [17,18,23,24] or willingness to continue use [29]. The use rate in these studies varied greatly. Notably, 86% of the participants expressed a willingness to engage with at least one component of the mHealth intervention in the future; however, actual use exhibited a marked decline over time [17,27]. Seven studies mentioned satisfaction and acceptance related to the interventions or mHealth devices [17,25,26,29-31,34,38]. Both the intervention and control groups exhibited high levels of satisfaction. Only 1 study [38] indicated that the satisfaction levels in the mHealth intervention group were significantly higher than those in the control group (Multimedia Appendix 4).

## Discussion

### Principal Findings

Among the 25 articles included in this review, we found 17 RCTs and 8 NRSIs with 2022 solid organ transplant recipients that met the criteria and evaluated the effectiveness of mHealth interventions in improving follow-up and management. The meta-analysis indicated that mHealth interventions can significantly enhance self-care ability, evidenced by an increase in the mean self-care ability score of 16.97 compared to the control group (intervention group: mean 250.14, SD 17.04 [14]; mean 238, SD 47.3 [17]; mean 106.58, SD 17.73 [42]; and mean 159.61, SD 9.29 [16]; control group: mean 228.80, SD 17.04 [14]; mean 232, SD 51.3 [17]; mean 83.77, SD 14.7 [42]; mean 145.14, SD 12.53 [16]). Most studies demonstrated improved medical regimen adherence, self-monitoring, and communication in the mHealth intervention groups relative to the control groups and affirmed that mHealth interventions enhance medication adherence among recipients, whereas most findings regarding medication blood concentrations revealed no significant differences between the intervention and control groups. A systematic review examining the effectiveness of mHealth self-management interventions in patients with hypertension revealed that approximately two-thirds of the studies reported enhancements in medication adherence and self-management behaviors whereas one-third of the studies did not demonstrate statistically significant improvements [46]. These results were similar to those of our study. The studies on mHealth interventions for physical activity were insufficient to draw definitive conclusions. Our meta-analysis demonstrated a nonsignificant reduction in all-cause mortality, as well as in rejection and infection rates, in the mHealth group compared to the control group. The meta-analysis revealed that the rehospitalization rate among recipients in the mHealth intervention group was significantly lower than that in the control group. However, no significant difference was observed in the outpatient visit rate between the 2 groups. Additionally, due to the limited number of studies examining the emergency department visit rate and the inconsistency of their findings, definitive conclusions regarding this outcome could not be drawn.

### mHealth Intervention Design and Features in This Review

Our review indicates that existing mHealth interventions for follow-up and management of transplant recipients predominantly focus on medication management and home monitoring and less on exercise management. Exercise management is vital for transplant recipients, especially lung and heart recipients. Numerous systematic reviews have demonstrated that exercise can substantially enhance physical function and support posttransplant rehabilitation in recipients, which is conducive to their long-term survival [47,48], which is conducive to posttransplant rehabilitation and the long-term survival of recipients. None of the studies in this review involved nutrition management after transplantation. Long-term nutritional management after transplantation is also critical to prevent adverse reactions worsened by immunosuppressive therapy, including diabetes, hypertension, dyslipidemia, obesity, and bone loss [49]. In the future, more mHealth intervention studies on exercise and nutrition management for solid organ transplant recipients should be carried out.

mHealth interventions in our review typically involved an integration of hardware devices and software apps. Commonly used devices included smartphones, tablets, smart robots, and smart pill boxes, all of which were equipped with wireless local area network, mobile network, or Bluetooth connectivity functions. Concurrently, mHealth apps are being continuously developed and implemented, enabling simultaneous connection to multiple mHealth devices and facilitating intervention in multiple aspects of follow-up management. Some research teams frequently refine the software they create to better match the continuously advancing mobile and monitoring technologies, enhancing the software’s intelligence, personalization, and comprehensive functionality for follow-up management. This is exemplified by the teams responsible for developing the Pocket Personal Assistant for Tracking Health for lung transplant recipients [14,17,18] and the Smartphone Medication Adherence Saves Kidneys app for kidney transplant recipients [31-33].

### Implementation Challenges and Recommendations

The included studies demonstrated high satisfaction among participants and usability of mHealth interventions and devices, and many participants also expressed a long-term willingness to use them, but the use rate of mHealth devices exhibited a notable decline over time. This decline can be attributed to several reasons, including technical feasibility (such as poor cellular signal, lack of wireless network at home, or data transmission delay due to server problems), device-related issues (such as loss, breakage, or excessive bulkiness), discomfort with use (such as the feeling of being monitored or finding the device itself to be stressful or worrisome), and user-related challenges (such as difficulties in operating the device or system, needing assistance to ensure appropriate setup and functionality, or being too busy). This is consistent with the findings of a mixed methods study on the telehealth experience among liver and kidney transplant recipients [50]. Transplant recipients’ acceptance and compliance with mHealth interventions are crucial for enhancing and sustaining health outcomes. The reliability of network infrastructure, the user-friendliness of mobile devices, and the requisite information security for mHealth interventions are very important. To address these challenges, it is recommended that, in addition to effective user training before the intervention, improvements are implemented in the following areas.

First, stakeholders, including transplant recipients, caregivers, and medical personnel at transplant follow-up centers, should be involved in all stages of the design and development of mHealth devices or apps to ensure that all designs meet the actual needs of the intended end users. Second, mHealth apps should be user-friendly and engaging, incorporating gamification elements and real-time feedback to enhance user interaction. In addition, the integration of emerging ITs into mHealth devices and apps presents additional opportunities [51]. Emerging technologies such as artificial intelligence can improve decision-making and personalize care, but they must address information security and privacy concerns, ensuring compliance with data protection laws and regulations.

### Limitations

There are some limitations to this study. First, the included studies varied in participant demographics, types of transplantation, sample size, mobile tools used, measurement tools, duration of the interventions, and assessment period, which can lead to heterogeneity and bias. Second, some outcomes depended on self-reports and the use of questionnaires, especially when assessments took place a long time after the intervention, which may lead to self-reporting bias and recall bias. Third, several studies featured notably small sample sizes, which could significantly affect the accuracy of estimates pertaining to the target population. Additionally, most studies were evaluated during the intervention period or immediately after the intervention. Only 4 studies evaluated outcomes 1 year or more after the intervention. Therefore, the subgroup analyses outlined in the protocol were not conducted, indicating a need for further evidence to substantiate the long-term effects of mHealth. Consequently, the findings of this systematic review should be interpreted with caution.

### Conclusions

In this systematic review, we conducted a narrative synthesis and meta-analysis of 23 RCTs and NRSIs. Our findings demonstrate that mHealth interventions exhibit varying effects across different outcomes in organ transplant recipients. Meta-analyses revealed statistically significant improvements in self-care ability and reductions in rehospitalization rates while showing no significant effects on all-cause mortality or complications. Through narrative synthesis, we identified potential benefits in several self-management behaviors, including medical regimen adherence, self-monitoring, communication and counseling behaviors, and medication adherence. However, these findings necessitate confirmation through future quantitative syntheses. Further research is required to elucidate the impact of mHealth interventions on physical activity, nutrition, and emergency and outpatient department visits among transplant recipients.

## Appendix

Multimedia Appendix 1 Search strategy.

Multimedia Appendix 2 Basic characteristics of the included studies.

Multimedia Appendix 3 Summary of outcomes.

Multimedia Appendix 4 Other outcomes.

Multimedia Appendix 5 Risk-of-bias assessment.

Multimedia Appendix 6 PRISMA (Preferred Reporting Items for Systematic Reviews and Meta-Analyses) checklist.

## Abbreviations

MeSH: Medical Subject Headings
NRSI: nonrandomized study of interventions
OR: odds ratio
PRISMA: Preferred Reporting Items for Systematic Reviews and Meta-Analyses
RCT: randomized controlled trial
