# Supplementary material for: Effectiveness of mHealth Interventions to Improve Follow-Up and Management Among Solid Organ Transplant Recipients: Systematic Review and Meta-Analysis
Source: JMIR Mhealth Uhealth. 2025 Dec 17;13:e69795. doi: 10.2196/69795 (PMC12756658; doi:10.2196/69795)
Supplement: Multimedia Appendix 1 [file mhealth_v13i1e69795_app1.docx]

**Multimedia Appendix 1 Search strategy**

**Search results for each source**

| **Database** | **No. refs found** | **Date searched** | **Searched by** |
| --- | --- | --- | --- |
| PubMed | 1713 | 17/6/2025 | X Lin |
| Web of Science Core Collection Databases | 2652 | 17/6/2025 | X Lin |
| Scopus | 2851 | 18/6/2025 | X Lin |
| Embase (Elsevier) | 2126 | 18/6/2025 | X Lin |
| CINAHL Plus with Full Text (EBSCOHost) | 739 | 17/6/2025 | X Lin |
| Cochrane Central Register of Controlled Trials (CENTRAL) | 3199 | 18/6/2025 | X Lin |

**Search Strategy for each source**

| **PubMed:** | |  |
| --- | --- | --- |
| Search Strategy: | |  |
| #1 | ("Transplantation"[MeSH Terms] OR "Organ Transplantation"[MeSH Terms] OR "Heart Transplantation"[MeSH Terms] OR "Liver Transplantation"[MeSH Terms] OR "Kidney Transplantation"[MeSH Terms] OR "Lung Transplantation"[MeSH Terms] OR "Pancreas Transplantation"[MeSH Terms] OR "Heart-Lung Transplantation"[MeSH Terms]) OR ((transplant*[Title/Abstract] OR graft*[Title/Abstract] OR allograft*[Title/Abstract] OR replacement[Title/Abstract] OR recipient*[Title/Abstract]) AND (organ[Title/Abstract] OR heart[Title/Abstract] OR cardiac[Title/Abstract] OR liver[Title/Abstract] OR hepatic[Title/Abstract] OR kidney[Title/Abstract] OR renal[Title/Abstract] OR lung[Title/Abstract] OR pulmonary[Title/Abstract] OR pancreas[Title/Abstract] OR pancreatic[Title/Abstract] OR multi-organ[Title/Abstract] OR combined[Title/Abstract] OR dual[Title/Abstract] OR "multiple organ"[Title/Abstract] OR simultaneous[Title/Abstract] OR sequential[Title/Abstract])) | 884477 |
| #2 | ((("mobile health"[Title/Abstract] OR "mHealth"[Title/Abstract] OR "m-health"[Title/Abstract] OR "m health"[Title/Abstract] OR "telemedicine"[MeSH Terms] OR "telemedicine"[Title/Abstract] OR "tele-medicine"[Title/Abstract] OR "telehealth"[Title/Abstract] OR "tele-health"[Title/Abstract] OR "eHealth"[Title/Abstract] OR "e-health"[Title/Abstract] OR "electronic health"[Title/Abstract] OR "telecare"[Title/Abstract] OR "tele-care"[Title/Abstract] OR “tele care”[Title/Abstract] OR "telenursing"[Title/Abstract] OR "tele-nursing"[Title/Abstract] OR "telemonitoring"[Title/Abstract] OR "tele-monitoring"[Title/Abstract] OR "remote monitoring"[Title/Abstract] OR "telerehabilitation*"[Title/Abstract] OR "tele-rehabilitation*"[Title/Abstract] OR "remote consultation"[MeSH Terms] OR "remote consult*"[Title/Abstract] OR "teleconsult*"[Title/Abstract] OR "tele-consult*"[Title/Abstract] OR "distance counseling"[MeSH Terms] OR "distance counsel*"[Title/Abstract] OR "digital health"[MeSH Terms] OR "digital health"[Title/Abstract] OR "digital medicine"[Title/Abstract] OR "digital therap*"[Title/Abstract] OR "Medical Informatics"[MeSH Terms] OR "medical informatics"[Title/Abstract] OR "health informatics"[Title/Abstract] OR "mobile technolog*"[Title/Abstract] OR "wireless technolog*"[Title/Abstract] OR "wireless technology"[MeSH Terms] OR "health information technolog*"[Title/Abstract] OR "HIT"[Title/Abstract]) OR ("smartphone"[MeSH Terms] OR "smartphone*"[Title/Abstract] OR "smart phone*"[Title/Abstract] OR "cell phone"[MeSH Terms] OR "cellphone*"[Title/Abstract] OR "cell phone*"[Title/Abstract] OR "mobile phone*"[Title/Abstract] OR "personal digital assistant*"[Title/Abstract] OR "PDA"[Title/Abstract] OR "tablet*"[Title/Abstract] OR "iPad*"[Title/Abstract] OR "smartwatch*"[Title/Abstract] OR "smart watch*"[Title/Abstract])) OR ("mobile device*"[Title/Abstract] OR "portable device*"[Title/Abstract] OR "wireless device*"[Title/Abstract] OR "handheld device*"[Title/Abstract] OR "wearable electronic devices"[MeSH Terms] OR "wearable*"[Title/Abstract] OR "wearable device*"[Title/Abstract] OR "fitness tracker*"[Title/Abstract] OR "activity tracker*"[Title/Abstract])) OR ("mobile applications"[MeSH Terms] OR "mobile application*"[Title/Abstract] OR "mobile health application*"[Title/Abstract] OR "mobile app*"[Title/Abstract] OR "health app*"[Title/Abstract] OR "mHealth app*"[Title/Abstract] OR "app"[Title/Abstract] OR "apps"[Title/Abstract] OR "WeChat"[Title/Abstract] OR "mini program*"[Title/Abstract] OR "software"[Title/Abstract]) | 1179823 |
| #3 | (((((((("follow-up"[Title/Abstract] OR "follow up"[Title/Abstract] OR "followup"[Title/Abstract] OR "aftercare"[MeSH Terms] OR "aftercare"[Title/Abstract] OR "continuity of care"[Title/Abstract]) OR ("self-management"[MeSH Terms] OR "self-management"[Title/Abstract] OR "self management"[Title/Abstract] OR "self care"[MeSH Terms] OR "self care"[Title/Abstract] OR "self-care"[Title/Abstract] OR "patient care management"[MeSH Terms])) OR ("rehabilitation"[MeSH Terms] OR "rehabilitation*"[Title/Abstract] OR "rehab"[Title/Abstract])) OR ("exercise"[MeSH Terms] OR "exercis*"[Title/Abstract] OR "physical activit*"[Title/Abstract] OR "physical train*"[Title/Abstract])) OR ("diet"[MeSH Terms] OR "diet*"[Title/Abstract] OR "nutrition"[MeSH Terms] OR "nutrition*"[Title/Abstract])) OR ("monitor*"[Title/Abstract] OR "patient monitoring"[MeSH Terms])) OR ("medication adherence"[MeSH Terms] OR "treatment adherence"[MeSH Terms] OR "adherenc*"[Title/Abstract] OR "complianc*"[Title/Abstract] OR "non-adherence*"[Title/Abstract] OR "nonadherence*"[Title/Abstract] OR "noncompliance*"[Title/Abstract] OR "non-compliance*"[Title/Abstract] OR "drug*"[Title/Abstract] OR "medication*"[Title/Abstract] OR "immunosuppress*"[Title/Abstract] OR "immunosuppressive agent*"[MeSH Terms] OR "immunosuppression"[MeSH Terms])) OR ("lifestyle*"[Title/Abstract] OR "life style*"[Title/Abstract] OR "health behavior"[MeSH Terms])) OR ("communication*"[Title/Abstract] OR "health communication"[MeSH Terms] OR "patient education"[MeSH Terms] OR "consult*"[Title/Abstract] OR "counsel*"[Title/Abstract] OR "patient counseling"[MeSH Terms] OR "health education"[MeSH Terms]) | 8526411 |
| #4 | #1 AND #2 AND #3 | 6861 |
| #5 | #4 AND (Filters applied: Adaptive Clinical Trial, Classical Article, Clinical Study, Clinical Trial, Comparative Study, Controlled Clinical Trial, Corrected and Republished Article, Equivalence Trial, Multicenter Study, Observational Study, Pragmatic Clinical Trial, Randomized Controlled Trial, Validation Study) | 1713 |

| **Web of Science Core Collection:** | |  |
| --- | --- | --- |
| Search Strategy: | |  |
| #1 | TS=((transplant* OR graft* OR allograft* OR replacement OR recipient*) AND (organ OR heart OR cardiac OR liver OR hepatic OR kidney OR renal OR lung OR pulmonary OR pancreas OR pancreatic OR multi-organ OR combined OR dual OR "multiple organ" OR simultaneous OR sequential)) | 498,920 |
| #2 | TS=(("mobile health" OR "mHealth" OR "m-health" OR "m health" OR "telemedicine" OR "tele-medicine" OR "telehealth" OR "tele-health" OR "eHealth" OR "e-health" OR "electronic health" OR "telecare" OR "tele-care" OR "telenursing" OR "tele-nursing" OR "telemonitoring" OR "tele-monitoring" OR "remote monitoring" OR "telerehabilitation*" OR "tele-rehabilitation*" OR "remote consult*" OR "teleconsult*" OR "tele-consult*" OR "distance counsel*" OR "digital health" OR "digital medicine" OR "digital therap*" OR "medical informatics" OR "health informatics" OR "mobile technolog*" OR "wireless technolog*" OR "health information technolog*" OR "HIT") OR ("smartphone*" OR "smart phone*" OR "cellphone*" OR "cell phone*" OR "mobile phone*" OR "personal digital assistant*" OR "PDA" OR "tablet*" OR "iPad*" OR "smartwatch*" OR "smart watch*") OR ("mobile device*" OR "portable device*" OR "wireless device*" OR "handheld device*" OR "wearable*" OR "wearable device*" OR "fitness tracker*" OR "activity tracker*") OR ("mobile application*" OR "mobile health application*" OR "mobile app*" OR "health app*" OR "mHealth app*" OR "app" OR "apps" OR "WeChat" OR "mini program*" OR "software")) | 1,061,526 |
| #3 | TS=(("follow-up" OR "follow up" OR "followup" OR "aftercare" OR "continuity of care") OR ("self-management" OR "self management" OR "self care" OR "self-care" OR "patient care management") OR ("rehabilitation*" OR "rehab") OR ("exercis*" OR "physical activit*" OR "physical train*") OR ("diet*" OR "nutrition*") OR ("monitor*" OR "patient monitoring") OR ("adherenc*" OR "complianc*" OR "non-adherence*" OR "nonadherence*" OR "noncompliance*" OR "non-compliance*" OR "drug*" OR "medication*" OR "immunosuppress*") OR ("lifestyle*" OR "life style*" OR "health behavior") OR ("communication*" OR "patient education" OR "consult*" OR "counsel*" OR "health education")) | 6,961,766 |
| #4 | #1 AND #2 AND #3 | 3282 |
| #5 | #4 AND (Document Types: Article) | 2652 |

| **Scopus:** | |  |
| --- | --- | --- |
| Search Strategy: | |  |
| #1 | ABS ( ( transplant* OR graft* OR allograft* OR replacement OR recipient* ) AND ( organ OR heart OR cardiac OR liver OR hepatic OR kidney OR renal OR lung OR pulmonary OR pancreas OR pancreatic OR multi-organ OR combined OR dual OR "multiple organ" OR simultaneous OR sequential ) ) | 568,843 |
| #2 | ABS ( ( "mobile health" OR "mHealth" OR "m-health" OR "m health" OR "telemedicine" OR "tele-medicine" OR "telehealth" OR "tele-health" OR "eHealth" OR "e-health" OR "electronic health" OR "telecare" OR "tele-care" OR "telenursing" OR "tele-nursing" OR "telemonitoring" OR "tele-monitoring" OR "remote monitoring" OR "telerehabilitation*" OR "tele-rehabilitation*" OR "remote consult*" OR "teleconsult*" OR "tele-consult*" OR "distance counsel*" OR "digital health" OR "digital medicine" OR "digital therap*" OR "medical informatics" OR "health informatics" OR "mobile technolog*" OR "wireless technolog*" OR "health information technolog*" OR "HIT" ) OR ( "smartphone*" OR "smart phone*" OR "cellphone*" OR "cell phone*" OR "mobile phone*" OR "personal digital assistant*" OR "PDA" OR "tablet*" OR "iPad*" OR "smartwatch*" OR "smart watch*" ) OR ( "mobile device*" OR "portable device*" OR "wireless device*" OR "handheld device*" OR "wearable*" OR "wearable device*" OR "fitness tracker*" OR "activity tracker*" ) OR ( "mobile application*" OR "mobile health application*" OR "mobile app*" OR "health app*" OR "mHealth app*" OR "app" OR "apps" OR "WeChat" OR "mini program*" OR "software" ) ) | 2,499,786 |
| #3 | ABS ( ( "follow-up" OR "follow up" OR "followup" OR "aftercare" OR "continuity of care" ) OR ( "self-management" OR "self management" OR "self care" OR "self-care" OR "patient care management" ) OR ( "rehabilitation*" OR "rehab" ) OR ( "exercis*" OR "physical activit*" OR "physical train*" ) OR ( "diet*" OR "nutrition*" ) OR ( "monitor*" OR "patient monitoring" ) OR ( "adherenc*" OR "complianc*" OR "non-adherence*" OR "nonadherence*" OR "noncompliance*" OR "non-compliance*" OR "drug*" OR "medication*" OR "immunosuppress*" ) OR ( "lifestyle*" OR "life style*" OR "health behavior" ) OR ( "communication*" OR "patient education" OR "consult*" OR "counsel*" OR "health education" ) ) | 11,799,453 |
| #4 | #1 AND #2 AND #3 | 3,384 |
| #5 | LIMIT-TO ( DOCTYPE , "ar" ) OR LIMIT-TO ( DOCTYPE , "cp" ) OR LIMIT-TO ( DOCTYPE , "sh" ) | 2,851 |

| **Embase:** | |  |
| --- | --- | --- |
| Search Strategy: | |  |
| #1 | (transplant*:ti,ab,kw OR graft*:ti,ab,kw OR allograft*:ti,ab,kw OR replacement:ti,ab,kw OR recipient*:ti,ab,kw) AND (organ:ti,ab,kw OR heart:ti,ab,kw OR cardiac:ti,ab,kw OR liver:ti,ab,kw OR hepatic:ti,ab,kw OR kidney:ti,ab,kw OR renal:ti,ab,kw OR lung:ti,ab,kw OR pulmonary:ti,ab,kw OR pancreas:ti,ab,kw OR pancreatic:ti,ab,kw OR 'multi organ':ti,ab,kw OR combined:ti,ab,kw OR dual:ti,ab,kw OR 'multiple organ':ti,ab,kw OR simultaneous:ti,ab,kw OR sequential:ti,ab,kw) AND [embase]/lim | 797,543 |
| #2 | ('mobile health':ti,ab,kw OR 'mhealth':ti,ab,kw OR 'm-health':ti,ab,kw OR 'm health':ti,ab,kw OR 'telemedicine':ti,ab,kw OR 'tele-medicine':ti,ab,kw OR 'telehealth':ti,ab,kw OR 'tele-health':ti,ab,kw OR 'ehealth':ti,ab,kw OR 'e-health':ti,ab,kw OR 'electronic health':ti,ab,kw OR 'telecare':ti,ab,kw OR 'tele-care':ti,ab,kw OR 'telenursing':ti,ab,kw OR 'tele-nursing':ti,ab,kw OR 'telemonitoring':ti,ab,kw OR 'tele-monitoring':ti,ab,kw OR 'remote monitoring':ti,ab,kw OR 'telerehabilitation*':ti,ab,kw OR 'tele-rehabilitation*':ti,ab,kw OR 'remote consult*':ti,ab,kw OR 'teleconsult*':ti,ab,kw OR 'tele-consult*':ti,ab,kw OR 'distance counsel*':ti,ab,kw OR 'digital health':ti,ab,kw OR 'digital medicine':ti,ab,kw OR 'digital therap*':ti,ab,kw OR 'medical informatics':ti,ab,kw OR 'health informatics':ti,ab,kw OR 'mobile technolog*':ti,ab,kw OR 'wireless technolog*':ti,ab,kw OR 'health information technolog*':ti,ab,kw OR 'hit':ti,ab,kw OR 'smartphone*':ti,ab,kw OR 'smart phone*':ti,ab,kw OR 'cellphone*':ti,ab,kw OR 'cell phone*':ti,ab,kw OR 'mobile phone*':ti,ab,kw OR 'personal digital assistant*':ti,ab,kw OR 'pda':ti,ab,kw OR 'tablet*':ti,ab,kw OR 'ipad*':ti,ab,kw OR 'smartwatch*':ti,ab,kw OR 'smart watch*':ti,ab,kw OR 'mobile device*':ti,ab,kw OR 'portable device*':ti,ab,kw OR 'wireless device*':ti,ab,kw OR 'handheld device*':ti,ab,kw OR 'wearable*':ti,ab,kw OR 'wearable device*':ti,ab,kw OR 'fitness tracker*':ti,ab,kw OR 'activity tracker*':ti,ab,kw OR 'mobile application*':ti,ab,kw OR 'mobile health application*':ti,ab,kw OR 'mobile app*':ti,ab,kw OR 'health app*':ti,ab,kw OR 'mhealth app*':ti,ab,kw OR 'app':ti,ab,kw OR 'apps':ti,ab,kw OR 'wechat':ti,ab,kw OR 'mini program*':ti,ab,kw OR 'software':ti,ab,kw) AND [embase]/lim | 772,087 |
| #3 | ('follow-up':ti,ab,kw OR 'follow up':ti,ab,kw OR 'followup':ti,ab,kw OR 'aftercare':ti,ab,kw OR 'continuity of care':ti,ab,kw OR 'self-management':ti,ab,kw OR 'self management':ti,ab,kw OR 'self care':ti,ab,kw OR 'self-care':ti,ab,kw OR 'patient care management':ti,ab,kw OR 'rehabilitation*':ti,ab,kw OR 'rehab':ti,ab,kw OR 'exercis*':ti,ab,kw OR 'physical activit*':ti,ab,kw OR 'physical train*':ti,ab,kw OR 'diet*':ti,ab,kw OR 'nutrition*':ti,ab,kw OR 'monitor*':ti,ab,kw OR 'patient monitoring':ti,ab,kw OR 'adherenc*':ti,ab,kw OR 'complianc*':ti,ab,kw OR 'non-adherence*':ti,ab,kw OR 'nonadherence*':ti,ab,kw OR 'noncompliance*':ti,ab,kw OR 'non-compliance*':ti,ab,kw OR 'drug*':ti,ab,kw OR 'medication*':ti,ab,kw OR 'immunosuppress*':ti,ab,kw OR 'lifestyle*':ti,ab,kw OR 'life style*':ti,ab,kw OR 'health behavior':ti,ab,kw OR 'communication*':ti,ab,kw OR 'patient education':ti,ab,kw OR 'consult*':ti,ab,kw OR 'counsel*':ti,ab,kw OR 'health education':ti,ab,kw) AND [embase]/lim | 8,353,049 |
| #4 | #1 AND #2 AND #3 | 7,300 |
| #5 | #4 AND ('article'/it OR 'article in press'/it OR 'conference paper'/it OR 'data papers'/it OR 'short survey'/it OR 'preprint'/it OR 'clinical trial'/it) | 2,126 |

| **CINAHL Plus with Full Text (EBSCOHost):** | |  |
| --- | --- | --- |
| Search Strategy: | |  |
| #1 | XB (transplant* OR graft* OR allograft* OR replacement OR recipient*) AND (organ OR heart OR cardiac OR liver OR hepatic OR kidney OR renal OR lung OR pulmonary OR pancreas OR pancreatic OR multi-organ OR combined OR dual OR "multiple organ" OR simultaneous OR sequential) | 77,720 |
| #2 | XB ("mobile health" OR "mHealth" OR "m-health" OR "m health" OR "telemedicine" OR "tele-medicine" OR "telehealth" OR "tele-health" OR "eHealth" OR "e-health" OR "electronic health" OR "telecare" OR "tele-care" OR "telenursing" OR "tele-nursing" OR "telemonitoring" OR "tele-monitoring" OR "remote monitoring" OR "telerehabilitation*" OR "tele-rehabilitation*" OR "remote consult*" OR "teleconsult*" OR "tele-consult*" OR "distance counsel*" OR "digital health" OR "digital medicine" OR "digital therap*" OR "medical informatics" OR "health informatics" OR "mobile technolog*" OR "wireless technolog*" OR "health information technolog*" OR "HIT") OR ("smartphone*" OR "smart phone*" OR "cellphone*" OR "cell phone*" OR "mobile phone*" OR "personal digital assistant*" OR "PDA" OR "tablet*" OR "iPad*" OR "smartwatch*" OR "smart watch*") OR ("mobile device*" OR "portable device*" OR "wireless device*" OR "handheld device*" OR "wearable*" OR "wearable device*" OR "fitness tracker*" OR "activity tracker*") OR ("mobile application*" OR "mobile health application*" OR "mobile app*" OR "health app*" OR "mHealth app*" OR "app" OR "apps" OR "WeChat" OR "mini program*" OR "software") | 176,975 |
| #3 | XB ("follow-up" OR "follow up" OR "followup" OR "aftercare" OR "continuity of care") OR ("self-management" OR "self management" OR "self care" OR "self-care" OR "patient care management") OR ("rehabilitation*" OR "rehab") OR ("exercis*" OR "physical activit*" OR "physical train*") OR ("diet*" OR "nutrition*") OR ("monitor*" OR "patient monitoring") OR ("adherenc*" OR "complianc*" OR "non-adherence*" OR "nonadherence*" OR "noncompliance*" OR "non-compliance*" OR "drug*" OR "medication*" OR "immunosuppress*") OR ("lifestyle*" OR "life style*" OR "health behavior") OR ("communication*" OR "patient education" OR "consult*" OR "counsel*" OR "health education") | 1,707,060 |
| #4 | #1 AND #2 AND #3 | 739 |

| **Cochrane Central Register of Controlled Trials (CENTRAL)** | |  |
| --- | --- | --- |
| Search Strategy: | |  |
| #1 | (transplant* OR graft* OR allograft* OR replacement OR recipient*) AND (organ OR heart OR cardiac OR liver OR hepatic OR kidney OR renal OR lung OR pulmonary OR pancreas OR pancreatic OR multi-organ OR combined OR dual OR "multiple organ" OR simultaneous OR sequential) in Title Abstract Keyword - in Trials (Word variations have been searched) | 70835 |
| #2 | ("mobile health" OR "mHealth" OR "m-health" OR "m health" OR "telemedicine" OR "tele-medicine" OR "telehealth" OR "tele-health" OR "eHealth" OR "e-health" OR "electronic health" OR "telecare" OR "tele-care" OR "telenursing" OR "tele-nursing" OR "telemonitoring" OR "tele-monitoring" OR "remote monitoring" OR "telerehabilitation*" OR "tele-rehabilitation*" OR "remote consult*" OR "teleconsult*" OR "tele-consult*" OR "distance NEXT counsel*" OR "digital health" OR "digital medicine" OR "digital NEXT therap*" OR "medical informatics" OR "health informatics" OR "mobile NEXT technolog*" OR "wireless NEXT technolog*" OR "health information NEXT technolog*" OR "HIT") OR ("smartphone*" OR "smart NEXT phone*" OR "cellphone*" OR "cell NEXT phone*" OR "mobile NEXT phone*" OR "personal digital NEXT assistant*" OR "PDA" OR "tablet*" OR "iPad*" OR "smartwatch*" OR "smart NEXT watch*") OR ("mobile NEXT device*" OR "portable NEXT device*" OR "wireless NEXT device*" OR "handheld NEXT device*" OR "wearable*" OR "wearable NEXT device*" OR "fitness NEXT tracker*" OR "activity NEXT tracker*") OR ("mobile NEXT application*" OR "mobile health NEXT application*" OR "mobile NEXT app*" OR "health NEXT app*" OR "mHealth NEXT app*" OR "app" OR "apps" OR "WeChat" OR "mini NEXT program*" OR "software") in Title Abstract Keyword - in Trials (Word variations have been searched) | 150081 |
| #3 | ("follow-up" OR "follow up" OR "followup" OR "aftercare" OR "continuity of care") OR ("self-management" OR "self management" OR "self care" OR "self-care" OR "patient care management") OR ("rehabilitation*" OR "rehab") OR ("exercis*" OR "physical NEXT activit*" OR "physical NEXT train*") OR ("diet*" OR "nutrition*") OR ("monitor*" OR "patient monitoring") OR ("adherenc*" OR "complianc*" OR "non-adherence*" OR "nonadherence*" OR "noncompliance*" OR "non-compliance*" OR "drug*" OR "medication*" OR "immunosuppress*") OR ("lifestyle*" OR "life NEXT style*" OR "health behavior") OR ("communication*" OR "patient education" OR "consult*" OR "counsel*" OR "health education") in Title Abstract Keyword - in Trials (Word variations have been searched) | 1345830 |
| #4 | #1 AND #2 AND #3 | 3199 |
