# Supplementary material for: Effectiveness of mHealth Interventions to Improve Follow-Up and Management Among Solid Organ Transplant Recipients: Systematic Review and Meta-Analysis
Source: JMIR Mhealth Uhealth. 2025 Dec 17;13:e69795. doi: 10.2196/69795 (PMC12756658; doi:10.2196/69795)
Supplement: Multimedia Appendix 2 [file mhealth_v13i1e69795_app2.docx]

**Appendix 2. Basic characteristics of the included studies**

| **Author, year, country** | **Study design** | **Participant, age range (I/C)** | **Sample size (I; C)** | **Type of intervention** | **mHealth tools and features** | **Intervention description** | **Duration of interventions** | **Assessment period** | **Outcomes** |
| --- | --- | --- | --- | --- | --- | --- | --- | --- | --- |
| DeVito Dabbs [1] 2009 , USA | RCT | Lung transplant recipients,  55±12.7 / 57±11.0 | 30  (15; 15) | Self-care | Pocket Personal Assistant for Tracking Health (Pocket PATH, a hand-held device) | Recipients were requested to enter data using Pocket PATH, review data trends by using the screens and graphs, and follow feedback instructions regarding reporting changes to their transplant coordinator. | 2 months | At the end of intervention | 1. Self-care agency 2. Self-care behaviors (Adhering to medical regimen, Self-monitoring, Communicating with coordinator) |
| DeVito Dabbs [4] 2016 , USA | RCT | Lung transplant recipients,  62 (51, 67) / 62 (51, 68) | 201  (99; 102) | Self-management | Pocket PATH program (uses a  smartphone platform) | Recipients received a smartphone with custom Pocket PATH programs to record daily health indicators, view graphical displays of trends, and receive automatic feedback messages advising them to notify the transplant coordinator if health indicators were critical. | 12 months | 2, 6, and 12 months during intervention | 1. Self-management behaviors (Self-monitoring, Adhering to medical regimen, Reporting critical health indicators) 2. Self-care agency 3. Re-hospitalization 4. All-cause mortality |
| Geramita [5] 2020 , USA | RCT | Lung transplant recipients,  56.2±12.3 / 56.0±14.2 | 105  (47; 58) | Long-  term adherence | A smartphone  with the Pocket PATH app | Authors recontacted participants from the previous study (DeVito Dabbs, 2016) over 2 years after its conclusion. Post-intervention, recipients kept the smartphone and could have continued to use the app which automatic decision support feature no longer functioned. | 12 months | More than 2 years after the end of the study | 1. Medical regimen adherence |
| Gomis-Pastor 2021 ,2023 [6,7] , Spain | RCT | Heart transplant recipients, 57±14 | 134  (71; 63) | Medication adherence | mHeart tool: a mobile and web-based software application | Multifaceted theory-based interventions were provided during the study period to optimize therapy management using the mHeart tool. | 1.6±0.6 years | At 6, 12 months during the intervention | 1. Medication adherence 2. CV% (coefficient of variation of concentrations) of immunosuppressive medication 3. Mortality, infection and complications 4. Re-hospitalization, emergency room visit, and primary care visits |
| Gonzales [8] , Fleming [9] 2021, USA | RCT | Kidney transplant recipients, 50±12 / 51±14 | 136  (68; 68) | Medication adherence | a mobile health app, integrated with risk-driven televisits and home-based BP and blood glucose monitoring | Recipients received clinical pharmacist–led supplemental medication therapy monitoring and management utilizing an app, including telemonitoring of medications, medical appointment adherence, weekly BP/glucose readings, and scheduling telehealth visits with participants. | 12 months | At the end of intervention | 1. Medication errors 2. Hospitalization 3. Infections 4. Tacrolimus intrapatient variability (IPV) |
| Han 2019 [10] , Korea | RCT | Kidney transplant recipients, 45 (35-54) / 43 (30-52) | 138  (71; 67) | Medication adherence | Adhere4U mobile medication manager application, a medication bottle with MEMS V prescription container lids | Adhere4U was provided to the recipients, which can provide audible and/or visual reminders, personal tracking data on missed and taken doses, patient’s medication adherence report. The MEMS lids recorded the time and date of bottle openings onto a digital chip, and data were downloaded. | 6 months | At 28, 90, 180 days during the intervention | 1. Medication adherence (nonadherence rate, self-reported adherence, visual analog scale) 2. Acute rejection episodes 3. Serum trough levels of the index medication |
| Henriksson [11] 2016, Sweden | RCT | Kidney transplant recipients, 44.3 (9-68) / 45.0 (2-69) | 80  (40; 40) | Medication adherence | a electronic medication dispenser (EMD) with cellular capabilities (tracking  device via Global System for Mobile Communications) | Recipients received a EMD. At the prescribed time for taking medication, the EMD gave visual and audible signals. If the patient did not take their medication, the audible signal was repeated with increasing frequency for 120 minutes. After this (or after the medication was taken), the EMD sent an SMS message to the web-based software. | 1 year | At the end of intervention | 1. Medication compliance 2. Outpatient follow-up visits 3. Hospital admissions 4. Rejection 5. Blood concentration of immunosuppressive medication |
| Hume [12] 2022, UK | RCT | Lung transplant recipients, 57 ± 9 / 58 ± 4 | 12  (7; 5) | Physical activity | a pedometer and a smartphone app which can provided daily feedback, encouragement, and educational messages | Patient uploads their activity data to the smartphone app via buletooth. Activity data is then transmitted to central databases. Patient receives feedback on their daily and weekly physical activity achievement and is allowed to contact with the researcher as required. | 12 weeks | At the end of intervention | 1. Physical activity (daily steps, movement intensity, time spent in sedentary and at least light activity) 2. Hospital admissions |
| Lee [13] 2019, USA | RCT | Liver transplant recipients, 58.5(51,65) / 60(56,65) | 100  (50; 50) | Home Monitoring | a telemedicine-based home management program (THMP): an electronic tablet and bluetooth devices | Recipients used smart tablet and peripherals to obtain and record vital signs measurements, get daily text messages, education videos, and video FaceTime. Tablet delivered daily questions and reminders based on postoperative day to assess clinical status. | 90 days | At the end of intervention | 1. 90-day hospital readmission rate |
| McGillicuddy [14] 2013, USA | RCT | Kidney transplant recipients, 42.44±12.04 / 57.60±8.28 | 19  (9;10) | Medication adherence | mHealth system: a smartphone, a wireless GSM electronic medication tray, a wireless bluetooth enabled Blood Pressure  monitor | Electronic medication tray provides customizable reminder signals. Text messages were sent to recipients every 3 days as a reminder to measure BP, and BP readings were automatically sent to a mobile phone and data repository. Patients were contacted when alerts indicated medication non-adherence, failure to measure BP, or that measured BP was outside of threshold ranges. | 3 months | At 1, 2, 3 months during the intervention | 1. Medication adherence 2. Blood pressure |
| McGillicuddy [15] 2015, USA | RCT | Kidney transplant recipients, 42.44 ±12.04 / 57.89 ±8.72 | 18  (9; 9) | Medication adherence | prototype mHealth system: Smartphone  Medication Adherence Saves Kidneys (SMASK) | Authors contacted 18 of the 19 participants who had completed the previous study (McGillicuddy et al, 2013) one year after the conclusion of the study. | 3 months | 12 months after completion of the study | 1. Systolic blood pressure |
| McGillicuddy [16] 2020, USA | RCT | Kidney transplant recipients, 52.1±11.3 / 51.5±12.5 | 82  (41; 41) | Medication adherence | an electronic  medication tray with reminder capabilities enabled, a bluetooth-enabled BP monitor, the SMASK smartphone app | At the dosing day and time, a blue light blinks. If the compartment remains unopened, an intermittent loud chime activates, then an automated reminder phone call or SMS text message is delivered to the participant. Participant was reminded via SMS messages to use the A&D Bluetooth BP monitor. The BP monitor interfaced with the SMASK app, and home-based BPs were transferred securely. | 6 months | 6 months after completion of the study | 1. Medication adherence 2. Tacrolimus trough variability |
| Sengpiel [17] 2010, Germany | RCT | Lung transplant recipients, 49.5 (33.3-55.8) / 48.5 (40.5-55.8) | 56  (28;28) | Home monitoring  (spirometry) | a bluetooth-capable AM1+ home spirometer , a synchronized  cell phone | Spirometry device automatically established a connection to the cell phone and a central database server. The highest measurement from 3 consecutive measurements was transmitted to a central database server within seconds of being measured. Patients whose FEV1<90% were instructed to contact the transplant center immediately. | 6 months | At the end of intervention | 1. Consultation 2. Adherence to home spirometry 3. Trough levels of immunosuppressive drugs 4. Hospitalizations, routine visits, emergency visits |
| Serper [24] 2020, USA | RCT | Kidney or liver transplant recipients, 50 ±15; 53±12; 54±13 | 127(42;44;41) | Physical activity | wearable trackers | Participants in Arm 2 and 3 (those with wearable trackers) had access to an online portal with health information including answers to health engagement questions as well as links with educational online resources regarding healthy diet and physical activity. Arm 3 received step goals and health engagement questions sent via text messages with financial incentives. | 12 weeks | 4 weeks after the intervention ended | 1. Physical activity (daily steps, weight change) |
| Tian [18] 2021, China | RCT | Liver transplant recipients, 43.35±10.44 / 48.00±8.68 | 102  (52; 50) | Home monitoring | Telemedicine follow-up management system: a intelligent service robot, doctor terminal app, patient terminal app, and management platforms | The transplant specialist remotely controlled the robot face-to-face communication with recipients approximately 20 minutes per day. Patients’ physiological parameters and feedback were telemonitored via the wireless equipment. The rehabilitation programs were administered with home-based video conference supervised exercise, and counseling. | 2 weeks | 1 year after completion of the study | 1. Readmission rate within 30 days after discharge 2. All-cause mortality 3. Complications (infection, rejection etc) |
| Levine [19] 2019, USA | NRSI | Kidney transplant recipients, 50/52/53 | 108  (20; 38; 50) | Medication adherence | a mobile app known as Transplant Hero, and/or a smart watch | 19% of patients received both the smart watch and mobile app (WMAU), 35% received the mobile app alone (MAU), and 46% received neither the mobile app nor the smart watch (NAU). App Transplant Hero® was utilized to function as an interactive alarm and to remind patients to take their medications as well as provide educational content. A Smart watch Technology® was used to display the reminder notiﬁcations. | 3 months | At 1, 3 months during the intervention | 1. Tacrolimus coefﬁcient of variability |
| Schenkel [20] 2020, UAS | NRSI | Lung transplant recipients, 54.4±12.7 / 55.2±11.9 | 56  (28;28) | Remote monitoring | a tablet(monitoring platforms system), bluetooth–enabled devices (measuring blood pressure, heart rate, weight, blood glucose, oxygen saturation, pulmonary function, and activity levels) | Bluetooth–enabled devices and tablets were delivered to patients. Tablets was used to report signs and symptoms, track appointments and medication compliance, and access educational videos and other materials. Monitoring platforms system leveraged Bluetooth technology to transmit patient vital sign measurements and respiratory parameters to transplant coordinators in real time, along with symptoms and activity levels. | 3 months | At the first two year after discharge | 1. Re-hospitalization (hospital readmissions, readmitted days, readmission charges) 2. Outpatient Visits 3. All-cause mortality |
| Tian [21] 2019, China | NRSI | Liver transplant recipients, 39±5 / 38±6 | 100  (50; 50) | Adherence to medical regimen | a telemedicine robot(can move independently and has a voice and video system, and the doctor can control and communicate through a computer or mobile app) | Participants received a telemedicine robot which can real-time remote monitor at regular intervals every day and has a health education column. The staff established online video communication with the recipient through the robot at a fixed time. A wechat group was established, and medical staff can answer any questions at any time. | 3 months | At the end of intervention | 1. Adherence to medical regimen 2. Duration per follow-up |
| Wickerson [22] 2023, Canada | NRSI | Lung, heart-lung, or lung-liver transplant recipients, 61 (54, 69) / 61 (56, 66) | 49  (23; 26) | Telerehabilitation | Vivify: a commercially available, customized, web-based remote care app | Telerehabilitation was delivered using Vivify to provide unsupervised home exercise that was guided asynchronously by physiotherapists. The same program of aerobic, resistance, and flexibility exercise for a minimum of 3 d/wk was used. A daily exercise prompt came up on the phone or tablet of the individual. Exercise responses were reviewed by physiotherapists and feedback was provided. | 3 months | At the end of intervention | 1. Physical activity (6-min walk test (6MWT), 5-time sit-to-stand (5STS), gait Speed, quadriceps torque (QT), Short Physical Performance Battery (SPPB)) |
| Xie [2] 2023, China | NRSI | Kidney transplant recipients | 160  (80; 80) | Self-management | a mobile application (Huawei Sports band) | The personal file of the recipients was established in the app, including medication, exercise, etc. The app contained 11 categories of health knowledge, provided intelligent self-monitoring forms to facilitate patients in recording important data, and provided automatic conversion, real-time online communication, medication and visit reminders functions. | NA | NA | 1. Self-management behavior |
| Yoo [3] 2021, Republic of Korea | NRSI | Heart transplant recipients, 50.57 ± 14.14 / 50.00 ± 13.19 | 56  (28; 28) | Self-care | a self-care health diary application (“health diary,” “today’s goal,” and “communication  space”) | Researchers received weekly on-line feedback from participants. The application provided participants with one-on-one counseling and encouraged self-care based on the activity records. Specific information on the application included medication, measurement, diet, activities and exercise, general healthcare, infection control, coping with an emergency, outpatient visit appointment date. | 4 weeks | At the end of intervention | 1. Self-efficacy 2. Self-care |
| Yang [25] 2022, China | NRSI | Kidney transplant recipients, 50.92 ± 10.37 / 51.73 ± 10.98 | 79  (39;40) | Self-management | "Internet +" programmatic follow-up platform: a remote management mobile APP("Flower of Life"), including recipients and medical staff We-chat ends | Medical staff and recipients established files on the mobile APP "Life Flower". Medical staff regularly informed the recipients of treatment suggestions and follow-up precautions. Related functions of APP included information query, follow-up reminder, disease warning, lifestyle guidance, transplantation education, online interaction and communication, self-monitoring, automatic statistical analysis of data | NA | 6 months after discharge | 1. Self-management ability |
| Zanetti-Yabur [23] 2017, USA | NRSI | Kidney or liver transplant recipients, 52.6 / 54.1 | 74  (21;53) | Medication adherence | a mobile phone application (Transplant  Hero™), an iPhone or iPad | Transplant Hero™ is an alarm system that alerts the user when it is time to take their medication. The software is an interactive, educational and simple to use tool that offers users positive reinforcement for medication adherence. | 3 months | At the end of intervention | 1. Medication adherence 2. Serum tacrolimus 3. Acute rejection |

RCT: randomized controlled trials

NRSI: non-randomized studies of interventions

NM: not mentioned

### **References**

1. DeVito Dabbs A, Dew MA, Myers B, et al. Evaluation of a hand-held, computer-based intervention to promote early self-care behaviors after lung transplant. Clin Transplant. 2009;23(4):537-545. PMID:19473201 doi:10.1111/j.1399-0012.2009.00992.x
2. Xie X, Wang X, Li A, et al. A Study of the Effectiveness of Mobile Health Application in A Self-management Intervention for Kidney Transplant Patients. Iran J Kidney Dis. 2023;17(5):263-270. doi: 10.52547/ijkd.7693
3. Yoo HJ, Suh EE. Effects of a smartphone-based self-care health diary for heart transplant recipients: A mixed methods study. Appl Nurs Res. 2021;58:151408. PMID:33745556 doi:10.1016/j.apnr.2021.151408
4. DeVito Dabbs A, Song MK, Myers BA, et al. A Randomized Controlled Trial of a Mobile Health Intervention to Promote Self-Management After Lung Transplantation. Am J Transplant. 2016;16(7):2172-2180. PMID:26729617 doi:10.1111/ajt.13701
5. Geramita EM, DeVito Dabbs AJ, DiMartini AF, et al. Impact of a Mobile Health Intervention on Long-term Nonadherence After Lung Transplantation: Follow-up After a Randomized Controlled Trial. Transplantation. 2020;104(3):640-651. PMID:31335759 doi:10.1097/TP.0000000000002872
6. Gomis-Pastor M, Mirabet Perez S, Roig Minguell E, et al. Mobile Health to Improve Adherence and Patient Experience in Heart Transplantation Recipients: The mHeart Trial. Healthcare (Basel). 2021;9(4):463. PMID:33919899 doi:10.3390/healthcare9040463
7. Gomis-Pastor M, Mirabet Perez S, De Dios Lopez A, et al. Does an eHealth Intervention Reduce Complications and Healthcare Resources? A mHeart Single-Center Randomized-Controlled Trial. J Cardiovasc Dev Dis. 2023;10(2):77. PMID:36826572 doi:10.3390/jcdd10020077
8. Gonzales HM, Fleming JN, Gebregziabher M, et al. Pharmacist-Led Mobile Health Intervention and Transplant Medication Safety: A Randomized Controlled Clinical Trial. Clin J Am Soc Nephrol. 2021;16(5):776-784. PMID:33931415 doi:10.2215/CJN.15911020
9. Fleming JN, Gebregziabher M, Posadas A, et al. Impact of a pharmacist-led, mHealth-based intervention on tacrolimus trough variability in kidney transplant recipients: A report from the TRANSAFE Rx randomized controlled trial. Am J Health Syst Pharm. 2021;78(14):1287-1293. PMID:33821958 doi:10.1093/ajhp/zxab157
10. Han A, Min SI, Ahn S, et al. Mobile medication manager application to improve adherence with immunosuppressive therapy in renal transplant recipients: A randomized controlled trial. PLoS One. 2019;14(11):e0224595. PMID:31689320 doi:10.1371/journal.pone.0224595
11. Henriksson J, Tydén G, Höijer J, Wadström J. A Prospective Randomized Trial on the Effect of Using an Electronic Monitoring Drug Dispensing Device to Improve Adherence and Compliance. Transplantation. 2016;100(1):203-209. PMID:26588006 doi:10.1097/TP.0000000000000971
12. Hume E, Muse H, Wallace K, et al. Feasibility and acceptability of a physical activity behavioural modification tele-coaching intervention in lung transplant recipients. Chron Respir Dis. 2022;19:14799731221116588. PMID:36306548 doi:10.1177/14799731221116588
13. Lee TC, Kaiser TE, Alloway R, et al. Telemedicine Based Remote Home Monitoring After Liver Transplantation: Results of a Randomized Prospective Trial. Ann Surg. 2019;270(3):564-572. PMID:31356267 doi:10.1097/SLA.0000000000003425
14. McGillicuddy JW, Gregoski MJ, Weiland AK, et al. Mobile Health Medication Adherence and Blood Pressure Control in Renal Transplant Recipients: A Proof-of-Concept Randomized Controlled Trial. JMIR Res Protoc. 2013;2(2):e32. PMID:24004517 doi:10.2196/resprot.2633
15. McGillicuddy JW, Taber DJ, Mueller M, et al. Sustainability of improvements in medication adherence through a mobile health intervention. Prog Transplant. 2015;25(3):217-223. PMID:26308780 doi:10.7182/pit2015975
16. McGillicuddy JW, Chandler JL, Sox LR, et al. Exploratory Analysis of the Impact of an mHealth Medication Adherence Intervention on Tacrolimus Trough Concentration Variability: Post Hoc Results of a Randomized Controlled Trial. Ann Pharmacother. 2020;54(12):1185-1193. PMID:32506922 doi:10.1177/1060028020931806
17. Sengpiel J, Fuehner T, Kugler C, et al. Use of telehealth technology for home spirometry after lung transplantation: a randomized controlled trial. Prog Transplant. 2010;20(4):310-317. PMID:21265282 doi:10.1177/152692481002000402
18. Tian M, Wang B, Xue Z, et al. Telemedicine for Follow-up Management of Patients After Liver Transplantation: Cohort Study. JMIR Med Inform. 2021;9(5):e27175. PMID:33999008 doi:10.2196/27175
19. Levine D, Torabi J, Choinski K, et al. Transplant surgery enters a new era: Increasing immunosuppressive medication adherence through mobile apps and smart watches. Am J Surg. 2019;218(1):18-20. PMID:30799019 doi:10.1016/j.amjsurg.2019.02.018
20. Schenkel FA, Barr ML, McCloskey CC, et al. Use of a Bluetooth tablet-based technology to improve outcomes in lung transplantation: A pilot study. Am J Transplant. 2020;20(12):3649-3657. PMID:32558226 doi:10.1111/ajt.16154
21. Tian B, Lu H, Zhang J, et al. Application of Telemedicine Robot in Follow-up After Liver Transplantation From Donation After Cardiac Death. Organ Transplantation. 2019;10(1):79-83. doi: 10.3969/j.issn.1674-7445.2019.01.012
22. Wickerson L, Rozenberg D, Singer LG, et al. Early Change in Lower Limb Strength and Function in Lung Transplant Patients After Center-Based and Telerehabilitation. J Cardiopulm Rehabil Prev. 2023;43(1):55-60. PMID:35961370 doi:10.1097/HCR.0000000000000728
23. Zanetti-Yabur A, Rizzo A, Hayde N, et al. Exploring the usage of a mobile phone application in transplanted patients to encourage medication compliance and education. Am J Surg. 2017;214(4):743-747. PMID:28256241 doi:10.1016/j.amjsurg.2017.01.026
24. Serper M, Barankay I, Chadha S, et al. A randomized, controlled, behavioral intervention to promote walking after abdominal organ transplantation: results from the LIFT study. Transpl Int. 2020;33(6):632-643. PMID:31925833 doi:10.1111/tri.13570
25. Yang JD, Song J, Zhu Q, Ye Q, Wu CZ. Management of "Internet plus" procedural follow-up program in renal transplant recipients. Chin J Gen Pract. 2022;20(7):1178-1181. doi:10.16766/j.cnki.issn.1674-4152.002552
