# Supplementary material for: Effectiveness of mHealth Interventions to Improve Follow-Up and Management Among Solid Organ Transplant Recipients: Systematic Review and Meta-Analysis
Source: JMIR Mhealth Uhealth. 2025 Dec 17;13:e69795. doi: 10.2196/69795 (PMC12756658; doi:10.2196/69795)
Supplement: Multimedia Appendix 3 [file mhealth_v13i1e69795_app3.docx]

**Appendix 3. Summary of Outcomes**

| **Primary Outcomes** | | **Study ID** | **Measurement tools and explanations** | **mHealth (IG)** | **Control (CG)** | **P-value** | **Effect** |
| --- | --- | --- | --- | --- | --- | --- | --- |
| Self-management abiliety and behaviors | Self-care ability | DeVito Dabbs 2009 | Perception of Self-Care Agency (PSCA), higher scores indicate higher self-care agency | M=250.14, SE=4.4 | M=228.8, SE=4.4 | *P*=.003 | Positive effect |
|  |  | DeVito Dabbs 2016 | Perception of Self-Care Agency (PSCA), higher scores indicate higher self-care agency (median, IQR) | 230 (212-245) (0-2 months) 233 (213-248) (2-6 months) 238 (216-251) (6-12 months) | 227 (207-245) (0-2 months) 229 (209-246) (2-6 months) 232 (209-247) (6-12 months) | *P*=.59 | No effect |
|  |  | Yoo 2021 | Self-care was measured with a tool developed specifically for HT recipients, a higher score means higher self-care (pre/post, P-value) | 142.36±12.83/159.61±9.29 (*P*<.001) | 140.11±15.60/145.14±12.54 (*P*=.147) | *P*<.003 | Positive effect |
|  |  | Yang 2022 | Self-management Ability Scale for Kidney Transplant Recipients | 77.66±13.22 (baseline)  106.58±17.73 (after the intervention) | 79.14±13.79 (baseline)  83.77±14.70 (after the intervention) | *P*=.628  *P*<.001 | No effect  Positive effect |
|  |  | Xie 2023 | Chronic kidney disease self-management behavior scale, higher scores indicate better self-management behavior | Scores of diet, treatment, repulsive body activity and psychosocial management in intervention group were significantly higher than the control group (*P* < .05) | | *P*<.05 | Positive effect |
|  | Medical regimen adherence | DeVito Dabbs 2009 | Health Habits Assessment, adherent to 9+areas means high adherence to the regimen | NA | NA | *P*<.025 | Positive effect |
|  |  | DeVito Dabbs 2016 | Health Habits Survey, high adherance was difined as median ≥ 8 | 67/89 (75%) (0-2 months) 44/78 (56%) (2-6 months) 31/74 (42%) (6-12 months) | 61/93 (66%) (0-2 months) 43/92 (47%) (2-6 months) 20/83 (24%) (6-12 months) | *P*=.05 | Positive effect |
|  |  | Geramita 2020 | Health Habits Survey, number of nonadherent elements | 2.7±1.5 | 3.1±1.5 | *P*=.218 | No effect |
|  |  | Tian 2019 | DCD liver transplant patient evaluation questionnaire, higher scores indicate higher adherence | 84.0±5.6 | 61.4±4.0 | *P*<.001 | Positive effect |
|  | Self-monitoring | DeVito Dabbs 2009 | Calculating the ratio of the number of days that patients actually recorded values for symptoms, temperature, or blood pressure to the number of days at home | NA | NA | *P*<.0125 | Positive effect |
|  |  | DeVito Dabbs 2016 | Calculated the percentage of days that LTRs performed self-monitorin | 52/90 (58%) (<25% ) 16/90 (18%) (25%-<50%) 22/90 (24%) (≥50% ) | 84/96 (88%) (<25% ) 5/96 (5%) (25%-<50%) 7/96 (7%) (≥50% ) |  | Positive effect |
|  |  | Sengpiel 2010 | Adherence to home spirometry (actual measurements/prescribed measurements); a median of measurements per patient; number of good adherence/moderate adherence/nonadherence | 97.2% (IQR, 73.9%-99.4%) 508 (377.5-521.3) 20/8/0 | 95.3% (IQR, 77.1%-99.5%) 488 (381.8-527.8) 21/6/1 | NA *P*=.068 NA | NA No effect No effect |
|  | Commnication and counseling | DeVito Dabbs 2009 | Patient initiated contacts to coordinator (M, SE) | NA | NA | *P*<.05 | Positive effect |
|  |  | Sengpiel 2010 | Time interval from the decline in FEV1 or the onset of symptoms to consultation(median, IQR); total number and average number per patient of consultation(n, median, IQR) | 70.0 (39.0-80.0) hours 258, 8.0 (6.3-11.8) times | 84.2 (29.7-100.0) 218, 8.0 (6.3-8.0) times | *P*=.60 *P*=.06 | No effect No effect |
|  |  | Tian 2019 | Duration per follow-up | 9±4 min | 13±4 min | *P*<.001 | Negative effect |
|  |  | DeVito Dabbs 2016 | Reporting critical indicators to the transplant coordinator (%, median, IQR) | 92.0%, 100 (91.6-100) | 56.4%, 77.5 (12.1-100) | *P*<.001 | Positive effect |
|  | Medication adherence | Gomis-Pastor 2021,2023 | Simpliﬁed Medication Adherence Questionnaire (SMAQ); Immunosuppressive Medication Timing Scale (IMTS); Basel Assessment of Adherence to Immunosuppressive Medications Scale (BAASIS); Haynes-Sackett questionnaire (percentage of adherence) | 85% (SMAQ) 89% (IMTS) 75% (BAASIS) 97% (Haynes Sackett) | 46% (SMAQ) 73% (IMTS) 64% (BAASIS) 84% (Haynes Sackett) | *P*<.001 *P*=.02 *P*=.227 *P*=.011 | Positive effect Positive effect No effect Positive effect |
|  |  | Han 2019 | Electronic monitoring (Nonadherence was defined as a taking adherence of<98% or >102% and/or at least one drug holiday); Basel Assessment ofAdherence to Immunosuppressive Medication Scale (BAASIS); visual analog scale (VAS) | 39/60 (65.0%) (non-adherent) 16/65 (BAASIS, 28d, non-adherent) 20/56 (BAASIS, 90d, non-adherent) 22/52 (BAASIS, 180d, non-adherent) 26/52 (VAS, 180d, non-adherent) | 36/58 (62.1%) (non-adherent) 24/62 (BAASIS, 28d, non-adherent) 31/58 (BAASIS, 90d, non-adherent) 30/54 (BAASIS, 180d, non-adherent) 30/54 (VAS, 180d, non-adherent) | *P*=.89 NA NA NA NA | No effect No effect Negative effect No effect No effect |
|  |  | Gonzales, Fleming 2021 | The incidence and severity of medication errors (n, M±SD), which were deﬁned as the participant taking a different medication than intended, on the basis of comparison of the EMR to the participant’s reported regimen | 614 (number of medication errors) 9.0±5.9 (medication error rate) | 1385 (number of medication errors) 20.4±14.0 (medication error rate) | *P*<.001 | Negative effect |
|  |  | Henriksson 2016 | Patients in the intervention group skipped their medicine dose on 524 of 23 820 occasions | 97.8% (compliance rate) | NA |  |  |
|  |  | McGillicuddy 2013 | Revised Russell et al's adherence socre calculation (0-1range, higher scores indicate better adherence) | 0.874 (mean, month 1) 0.929 (mean, month 2) 0.945 (mean, month 3) | 0.533 (mean, month 1) 0.587 (mean, month 2) 0.574 (mean, month 3) | *P*<.05 | Positive effect |
|  |  | McGillicuddy 2020 | Calculated using timestamps of openings of Vaica pill tray compartments (medication doses taken within a 3-hour window received a full score, within a 3 to 6-hour window received partial credit, and outside 6-hour windows did not count for any adherence credit) | 89.8% (month 1) 90.1% (month 3) 88.6% (month 6) | 45.3% (month 1) 38.5% (month 3) 45.7% (month 6) | *P*<.001 *P*<.001 *P*<.001 | Positive effect Positive effect Positive effect |
|  |  | Zanetti-Yabur 2017 | Medication adherence scale, higher scores indicate better adherence; Immunosuppression Assessment Test (IAT), the IAT assigns 1-point for each correct medication with a maximum of 3-points | 0.84 (Medication adherence scale) 2.25 (IAT) | 0.74 (Medication adherence scale) 1.73 (IAT) | NA *P*=.19 | No effect No effect |
|  | Immunosuppressive medication blood concentration and its coefficient of variation (CV), intrapatient variability (IPV) | Gomis-Pastor 2021,2023 | Variability for tacrolimus and cyclosporine blood levels was assessed by the coefﬁcient of variation of concentrations (CV% = (SD/µ) × 100) for each patient. CV% > 30% were interpreted as nonadherent | 41% (percentage of patients with CV%>30%) | 47% (percentage of patients with CV%>30%) | *P*=.526 | No effect |
|  |  | McGillicuddy 2020 | An intrapatient 12-month rolling average of the coefficient of variation ([CV]; calculated as [Mean/SD] × 100%). A cutpoint of 40% was used to define normal versus high variability. | Patients in SMASK intervention arm had a significant reduction in the mean 12-month rolling average of the CV and had a significant improvement in the proportion of patients achieving an intrapatient tacrolimus CV <40%. | | *P*=.046 *P*=.001 | Negative effect Positive effect |
|  |  | Levine 2019 | coefﬁcient of variability (CV) = (standard deviation/mean tacrolimus)*100 | 35.5% (WMAU) 30.4% (MAU) (month1) 33.8% (WMAU) 33.0% (MAU) (month3) | 31.7% (NAU) (month 1) 32.8% (NAU) (month 3) | *P*_W-N1_=.96 *P*_M-N1_=.68 *P*_W-N3_=.81 *P*_M-N3_=.45 | All no effect |
|  |  | Gonzales, Fleming 2021 | IPVs of 30% or greater likely indicated patients with clinical nonadherence | IG demonstrated a statistically significant decrease in tacrolimus IPV over time as compared to CG (P = 0.0133). | | *P*=.0133 | Negative effect |
|  |  | Han 2019 | Intraindividual variability during the study period, (median, IQR) | 12.5 (8.2–18.4) | 13.3 (8.4–18.7) | *P*=.906 | No effect |
|  |  | Henriksson 2016 | Tacrolimus concentration | NA | NA | NA | No effect |
|  |  | Sengpiel 2010 | Trough levels of immunosuppressive drugs in target range (median, IQR) (Target levels were 230 to 280 ng/mL for cyclosporine and 10 to 15 ng/mL for tacrolimus) | 34.1% (19.7%-40.8%) | 34.0% (20.3%-43.0%) | *P*=.87 | No effect |
|  |  | Zanetti-Yabur 2017 | Serum Tacrolimus Level | 9.5 Ug/L | 9.5 Ug/L | *P*=.93 | No effect |
|  | Physical activity | Hume 2022 | Daily steps(steps/day); movement intensity(VMU); time spent in sedentary activity(min/day); time spent in at least light activity(min/day) (within group change) | 3475±3422 (daily steps) 153±166 (movement intensity) -57±128 (sedentary activity) 37±24 (at least light activity) | 1159±991 (daily steps) -3±61 (movement intensity) 22±52 (sedentary activity) 3±37 (at least light activity) | *P*=.089 *P*=.037 *P*=.114 *P*=.040 | No effect Positive effect No effect Positve effect |
|  |  | Wickerson 2023 | 6-min walk test (6MWT); 5-time sit-to-stand (5STS) change; gait speed; quadriceps torque (QT); Short Physical Performance Battery (SPPB) (pre-post change, median, IQR, P-value) | 106 (11,132) *P*=.02 0.26 (-1.23, 3.31) *P*=.39 0.21 (0.11, 0.47) *P*<.001 -9.6 (-29, -2.3) *P*=.02 0 (-1,0) *P*=.25 | 54 (35, 102) *P*=.001 0.84 (-0.15, 2.2) *P*=.08 0.04 (-0.08, 0.35) *P*=.13 -1.6 (-2.5, 8.1) *P*=.79 0 (0, 0) *P*=0.18 | NA | NA |
|  |  | Serper 2020 | Daily steps throughout study period (mean, SD); weight change(kg, mean, SD) | NA (control group)  1.0(3.9)(control group) | 7045(3296)(control with device group),7691(2978)(intervention group)  2.7(5.3)(control with device group), 0.81(4.0)(intervention group) | *P*=.07  *P*=.30 | No effect  No effect |
| Health outcomes | All-cause mortality | DeVito Dabbs 2016 |  | 11/99 (11.1%) | 8/102 (7.8%) | *P*=.25 | No effect |
|  |  | Gomis-Pastor 2021,2023 |  | 3/71 (4.2%) | 6/63 (9.5%) | *P*=.4 | No effect |
|  |  | Tian 2021 | Mortality at 1 year | 3/52 (5.8%) | 5/50 (10.0%) | *P*=.50 | No effect |
|  |  | Schenkel 2020 | Mortality at 2 years | 2/28 (7.1%) | 6/28 (21.4%) | *P*=.252 | No effect |
|  | Complications | Gomis-Pastor 2021,2023 | Infection (n, %); other complication | 5/29 (17.2%) (infection) Except for "infection" and "cardiovascular events", which were significantly lower in the IG group than in the CG group, there was no significant difference in the incidence of other complications between the two groups | 17/30 (56.7%) (infection) | *P*=.03 | Negative effect Two negative effect, mostly no effect |
|  |  | Tian 2021 | Infection (n, %); rejection (n, %); other complication | 2/52 (3.9%) (infection) 4/52 (7.7%) (rejection) Occurrences of significant complications, such as primary graft failure, primary graft dysfunction, acute rejection reaction, vascular complications, biliary complications, tumor recurrence, and severe infection, did not differ significantly between the two groups | 2/50 (4.0%) (infection) 3/50 (6.0%) (rejection) | *P*=.97 *P*=.75 | No effect No effect No effect |
|  |  | Gonzales, Fleming 2021 | Infection rate (infections per patient-year, 95%CI) | 1.32 (0.92-1.74) | 1.26 (0.76-1.76) | *P*=.75 | No effect |
|  |  | Han 2019 | Rejection (n, %) | 2/69 (2.9%) | 1/67 (1.5%) | *P*=1.000 | No effect |
|  |  | Henriksson 2016 | Number of the patients and rejection events | 4 (number of the patients) 6 (number of the rejection events) | 13 (number of the patients) 27 (number of the rejection events) | *P*=.019 | Negative effect |
|  |  | Zanetti-Yabur 2017 | Rejection (n, %) | 1/21 | 4/53 | *P*=.86 | No effect |
| Health Resourse Use | Re-hospitalization | DeVito Dabbs 2016 | n/N (%); total rehospital days (median, IQR) | 80/99 (81%) 12(3-32) days | 87/102 (85%) 14.5 (4-47) days | *P*=.51 *P*=.60 | No effect No effect |
|  |  | Gomis-Pastor 2021,2023 | n/N (%) | 23/71 (32.4%) | 33/58 (56.9%) | *P*=.004 | Negative effect |
|  |  | Gonzales, Fleming 2021 | Hospitalization rate (hospitalizations per patient-year, 95%CI) | 0.65 (0.40-0.94) | 1.08 (0.60-1.6) | *P*=.007 | Negative effect |
|  |  | Henriksson 2016 | n/N (%) | 22/40 | 31/40 | *P*=.854 | No effect |
|  |  | Hume 2022 | n/N (%) | 4/7 | 2/5 | NA | NA |
|  |  | Lee 2019 | n/N (%), 90-day hospital readmissions | 14/50 (28%) | 29/50 (58%) | *P*=.004 | Negative effect |
|  |  | Sengpiel 2010 | n/N (%) | 16/28 | 15/28 | *P*=.99 | No effect |
|  |  | Tian 2021 | Readmission rate within 30 days after discharge | 0.08±0.27 | 0.24±0.43 | *P*=.02 | Negative effect |
|  |  | Schenkel 2020 | hospital readmissions; readmitted days; readmission charges(in thousands) (Events, Events Per Person Per Year) | 66, 1.27 543, 10.41 7562, 145.04 | 112, 2.26 1116, 22.50 13748, 277.14 | *P*<.001 *P*<.001 *P*<.001 | Negative effect Negative effect Negative effect |
|  | Emergency department visits | Gomis-Pastor 2021,2023 | n/N (%) | 36/71 (50.7%) | 40/58 (69.0%) | *P*=.03 | Negative effect |
|  |  | Sengpiel 2010 | Number of emergency consultations per patient | 1.0(0.0-3.0) | 1.0(0.0-2.0) | *P*=.68 | No effect |
|  | Outpatient visits/Primary care visits | Gomis-Pastor 2021,2023 | Primary care visits, n/N (%) | 62/71 (87.3%) | 55/58 (94.8%) | *P*=.1 | No effect |
|  |  | Henriksson 2016 | Outpatient follow-up visits (total times of missed outpatient follow-up visits) | 8 | 3 | NA | No effect |
|  |  | Sengpiel 2010 | Number of routine consultations per patient | 7.0(6.0-9.0) | 6.0(6.0-7.0) | *P*=.24 | No effect |
|  |  | Schenkel 2020 | Mean Outpatient Visits in 2 Yr (SD) | 24.0±4.8 | 24.7±8.3 | *P*=.723 | No effect |

### **References**

1. DeVito Dabbs A, Dew MA, Myers B, et al. Evaluation of a hand-held, computer-based intervention to promote early self-care behaviors after lung transplant. Clin Transplant. 2009;23(4):537-545. PMID:19473201 doi:10.1111/j.1399-0012.2009.00992.x
2. Xie X, Wang X, Li A, et al. A Study of the Effectiveness of Mobile Health Application in A Self-management Intervention for Kidney Transplant Patients. Iran J Kidney Dis. 2023;17(5):263-270. doi: 10.52547/ijkd.7693
3. Yoo HJ, Suh EE. Effects of a smartphone-based self-care health diary for heart transplant recipients: A mixed methods study. Appl Nurs Res. 2021;58:151408. PMID:33745556 doi:10.1016/j.apnr.2021.151408
4. DeVito Dabbs A, Song MK, Myers BA, et al. A Randomized Controlled Trial of a Mobile Health Intervention to Promote Self-Management After Lung Transplantation. Am J Transplant. 2016;16(7):2172-2180. PMID:26729617 doi:10.1111/ajt.13701
5. Geramita EM, DeVito Dabbs AJ, DiMartini AF, et al. Impact of a Mobile Health Intervention on Long-term Nonadherence After Lung Transplantation: Follow-up After a Randomized Controlled Trial. Transplantation. 2020;104(3):640-651. PMID:31335759 doi:10.1097/TP.0000000000002872
6. Gomis-Pastor M, Mirabet Perez S, Roig Minguell E, et al. Mobile Health to Improve Adherence and Patient Experience in Heart Transplantation Recipients: The mHeart Trial. Healthcare (Basel). 2021;9(4):463. PMID:33919899 doi:10.3390/healthcare9040463
7. Gomis-Pastor M, Mirabet Perez S, De Dios Lopez A, et al. Does an eHealth Intervention Reduce Complications and Healthcare Resources? A mHeart Single-Center Randomized-Controlled Trial. J Cardiovasc Dev Dis. 2023;10(2):77. PMID:36826572 doi:10.3390/jcdd10020077
8. Gonzales HM, Fleming JN, Gebregziabher M, et al. Pharmacist-Led Mobile Health Intervention and Transplant Medication Safety: A Randomized Controlled Clinical Trial. Clin J Am Soc Nephrol. 2021;16(5):776-784. PMID:33931415 doi:10.2215/CJN.15911020
9. Fleming JN, Gebregziabher M, Posadas A, et al. Impact of a pharmacist-led, mHealth-based intervention on tacrolimus trough variability in kidney transplant recipients: A report from the TRANSAFE Rx randomized controlled trial. Am J Health Syst Pharm. 2021;78(14):1287-1293. PMID:33821958 doi:10.1093/ajhp/zxab157
10. Han A, Min SI, Ahn S, et al. Mobile medication manager application to improve adherence with immunosuppressive therapy in renal transplant recipients: A randomized controlled trial. PLoS One. 2019;14(11):e0224595. PMID:31689320 doi:10.1371/journal.pone.0224595
11. Henriksson J, Tydén G, Höijer J, Wadström J. A Prospective Randomized Trial on the Effect of Using an Electronic Monitoring Drug Dispensing Device to Improve Adherence and Compliance. Transplantation. 2016;100(1):203-209. PMID:26588006 doi:10.1097/TP.0000000000000971
12. Hume E, Muse H, Wallace K, et al. Feasibility and acceptability of a physical activity behavioural modification tele-coaching intervention in lung transplant recipients. Chron Respir Dis. 2022;19:14799731221116588. PMID:36306548 doi:10.1177/14799731221116588
13. Lee TC, Kaiser TE, Alloway R, et al. Telemedicine Based Remote Home Monitoring After Liver Transplantation: Results of a Randomized Prospective Trial. Ann Surg. 2019;270(3):564-572. PMID:31356267 doi:10.1097/SLA.0000000000003425
14. McGillicuddy JW, Gregoski MJ, Weiland AK, et al. Mobile Health Medication Adherence and Blood Pressure Control in Renal Transplant Recipients: A Proof-of-Concept Randomized Controlled Trial. JMIR Res Protoc. 2013;2(2):e32. PMID:24004517 doi:10.2196/resprot.2633
15. McGillicuddy JW, Taber DJ, Mueller M, et al. Sustainability of improvements in medication adherence through a mobile health intervention. Prog Transplant. 2015;25(3):217-223. PMID:26308780 doi:10.7182/pit2015975
16. McGillicuddy JW, Chandler JL, Sox LR, et al. Exploratory Analysis of the Impact of an mHealth Medication Adherence Intervention on Tacrolimus Trough Concentration Variability: Post Hoc Results of a Randomized Controlled Trial. Ann Pharmacother. 2020;54(12):1185-1193. PMID:32506922 doi:10.1177/1060028020931806
17. Sengpiel J, Fuehner T, Kugler C, et al. Use of telehealth technology for home spirometry after lung transplantation: a randomized controlled trial. Prog Transplant. 2010;20(4):310-317. PMID:21265282 doi:10.1177/152692481002000402
18. Tian M, Wang B, Xue Z, et al. Telemedicine for Follow-up Management of Patients After Liver Transplantation: Cohort Study. JMIR Med Inform. 2021;9(5):e27175. PMID:33999008 doi:10.2196/27175
19. Levine D, Torabi J, Choinski K, et al. Transplant surgery enters a new era: Increasing immunosuppressive medication adherence through mobile apps and smart watches. Am J Surg. 2019;218(1):18-20. PMID:30799019 doi:10.1016/j.amjsurg.2019.02.018
20. Schenkel FA, Barr ML, McCloskey CC, et al. Use of a Bluetooth tablet-based technology to improve outcomes in lung transplantation: A pilot study. Am J Transplant. 2020;20(12):3649-3657. PMID:32558226 doi:10.1111/ajt.16154
21. Tian B, Lu H, Zhang J, et al. Application of Telemedicine Robot in Follow-up After Liver Transplantation From Donation After Cardiac Death. Organ Transplantation. 2019;10(1):79-83. doi: 10.3969/j.issn.1674-7445.2019.01.012
22. Wickerson L, Rozenberg D, Singer LG, et al. Early Change in Lower Limb Strength and Function in Lung Transplant Patients After Center-Based and Telerehabilitation. J Cardiopulm Rehabil Prev. 2023;43(1):55-60. PMID:35961370 doi:10.1097/HCR.0000000000000728
23. Zanetti-Yabur A, Rizzo A, Hayde N, et al. Exploring the usage of a mobile phone application in transplanted patients to encourage medication compliance and education. Am J Surg. 2017;214(4):743-747. PMID:28256241 doi:10.1016/j.amjsurg.2017.01.026
24. Serper M, Barankay I, Chadha S, et al. A randomized, controlled, behavioral intervention to promote walking after abdominal organ transplantation: results from the LIFT study. Transpl Int. 2020;33(6):632-643. PMID:31925833 doi:10.1111/tri.13570
25. Yang JD, Song J, Zhu Q, Ye Q, Wu CZ. Management of "Internet plus" procedural follow-up program in renal transplant recipients. Chin J Gen Pract. 2022;20(7):1178-1181. doi:10.16766/j.cnki.issn.1674-4152.002552
