# Supplementary material for: Effectiveness of mHealth Interventions to Improve Follow-Up and Management Among Solid Organ Transplant Recipients: Systematic Review and Meta-Analysis
Source: JMIR Mhealth Uhealth. 2025 Dec 17;13:e69795. doi: 10.2196/69795 (PMC12756658; doi:10.2196/69795)
Supplement: Multimedia Appendix 4 [file mhealth_v13i1e69795_app4.docx]

**Appendix 4. Other Outcomes**

| **Other Outcomes** | **Study ID** | **Type of evaluation** | **mHealth** | **Control** | **P-value** |
| --- | --- | --- | --- | --- | --- |
| Retention rate | Gonzales, Fleming 2021 | Two participants withdrew from the study intervention arm before completing the study for a 99% retention rate. | 99.0% |  |  |
|  | Han 2019 | The 6-month intervention was completed by 54 (80.6%) patients in the control group and 52 (73.2%) patients in the mobile group. Dropouts were mainly because of issues related to the MEMS bottle (loss or breakage, n = 9; discomfort with use, n = 13). | 73.2% | 80.6% |  |
|  | Henriksson 2016 | Six of the 40 participants in the intervention group withdrew from the study prematurely, 3 of them due to “a feeling of being monitored.” One participant experienced the EMD to be extremely stressful/worrisome, which resulted in the withdrawal from the study. None of the participants in the control group withdrew from the study prematurely. | 85.0% | 100.0% |  |
|  | Hume 2022 | The retention rate was 86% for patients that consented to take part in the study. The dropout rates were equal between the tele-coaching and usual care group. | 87.5% | 83.3% | No effect |
|  | McGillicuddy 2013 | There were 6 subjects did not complete the lead-in phase, 5 for technical reasons relating to poor cellular signal at their home. |  |  |  |
|  | McGillicuddy 2020 | 82 were randomized and 71 completed the 6-month study. 8 participants in mHealth intervention arm were withdrawn due to non-comliance of study protocol, request by subject to inability to use technology or busy home life, while 3 participants in control arm were withdrawn due to non-compliance of study protocol. | 80.5% | 92.7% |  |
|  | Serper 2020 | The study retention rate was 117/127 participants (92.1%). | 90.9%(control with device group),87.8%(intervention group) | 97.6% |  |
|  | Tian 2021 | A total of 60 patients were eligible for inclusion in the telemedicine group, but 6 patients were excluded from the study because they did not have a wireless network at home, 2 patients did not start the program because they could not use the telemedicine follow-up management system. | 86.7% | 100.0% |  |
| Adoption and utilization | DeVito Dabbs 2016 | The frequency of participants who did not self-monitor at 0–2, 2–6, and 6–12 months were 8 (9%) of 88, 17 (18%) of 92, and 25 (28%) of 90, respectively, for the Pocket PATH group and 46 (48%) of 96, 64 (66%) of 97, and 74 (77%) of 96, respectively, for the control group. | 91% (0-2 months) 82% (2-6 months) 72% (6-12 months) | 52% (0-2 months) 34% (2-6 months) 26% (6-12 months) |  |
|  | Gomis-Pastor 2021-2023 | At the end of the study, 85% of the patients assigned to mHeart were engaged on a daily basis, but 10% of them needed to be reminded at least once during the study period to use the mHeart platform. None of the participants completely stopped using mHeart. | 85.0% |  |  |
|  | Han 2019 | Based on the use of the medication reminder function in the mobile group was low, with a rate of 47.6% at visit 1 (28 days), 33.9% at visit 2 (90 days), and 11.5% at visit 3 (180 days). | 47.6 (28days) 33.9% (90days) 11.5% (180days) |  |  |
|  | Hume 2022 | Usage of the pedometer was excellent, with all patients wearing it for over 90% of days and rating the pedometer and telephone contact as the most vital aspects. Importantly, 86% of patients were willing to use at least one aspect of the intervention in the future. | 100.0% | 100.0% |  |
|  | Lee 2019 | Participation and adherence with telemedicine was 86% for basic health sessions (vital sign recording), but only 6% for using messaging or Face-Time. Only 60% of the patients would respond to text messages in the first month and this dropped to 25%. 70% of patients used phone calls for issues , and 75% viewed educational video slides. |  |  |  |
|  | Schenkel 2020 | All patients enrolled in monitoring complied with the program, reporting data at least weekly during follow-up, and 53.5% of monitored patients reported data three times per week or more. |  |  |  |
|  | Yoo 2021 | During the 4 weeks, average login days of 28 intervention participants was 22.0 days, total login frequency was 36.2 times, login duration was 86.9 minutes, frequency of using health quizzes was 5.3 times, and frequency of communication with the transplant team was 3.8 times. |  |  |  |
|  | Geramita 2020 | Only 2 of the 47 participants in the intervention group were using Pocket PATH at follow-up period. | 4.3% (2years after intervention) |  |  |
| Satisfaction and acceptance | DeVito Dabbs 2016 | When asked at the end of the study, 100% of participants were satisﬁed with the treatment condition to which they were randomized. | 100.0% | 100.0% |  |
|  | Gonzales, Fleming 2021 | In terms of the app itself, 93% of respondents indicated that they were either satisfied (n = 15 [24%]) or very satisfied (n = 43[69%]) with the ease of use of the app. | 93.0% |  |  |
|  | Hume 2022 | Tele-coaching was well accepted by patients, with 86% indicating that they enjoyed taking part. Furthermore, 86% of patients reported that the intervention “helped them a lot” to improve their physical activity levels, with 86% of patients indicating that the smartphone app was either “very easy” or “easy” to use. | 86.0% |  |  |
|  | Lee 2019 | Patients randomized to the THMP group were receptive to the device, but assistance was needed at the home to ensure appropriate hookup and function of the remote device in nearly one-third of patients (32%). |  |  |  |
|  | McGillicuddy 2013 | The acceptability of patients’ participation in either the mHealth or standard care protocol was high with 75% (41/55) , with 8 refusing over concerns that either the electronic medication tray was “too bulky”, that they were “too busy”, or that they would have to travel too much. The mHealth group reported high overall satisfaction with the mHealth system (average score 4.8/5 point). The mHealth system was easy for the subjects to learn to use (4.7/5) and easy to use in their home (4.8/5). They also found the system useful for medication and health management (4.3/5). |  |  |  |
|  | Sengpiel 2010 | 18 of 21 patients (85.8% ) in the Bluetooth group and 16 of 19 patients in the home spirometry alone group (84.2%; *P*=.31) perceived the medical care as “very good” or “good.” | 85.7% | 84.2% | *P*=.31 |
|  | Tian 2019 | The satisfaction of liver transplant recipients in the intervention group was (19.8±2.6), which was significantly higher than that in the control group (16.2±3.1) (*t*=6.234, *P*<.001). | 19.8±2.6 | 16.2±3.1 | *P*<.001 |

### **References**

1. Yoo HJ, Suh EE. Effects of a smartphone-based self-care health diary for heart transplant recipients: A mixed methods study. Appl Nurs Res. 2021;58:151408. PMID:33745556 doi:10.1016/j.apnr.2021.151408
2. DeVito Dabbs A, Song MK, Myers BA, et al. A Randomized Controlled Trial of a Mobile Health Intervention to Promote Self-Management After Lung Transplantation. Am J Transplant. 2016;16(7):2172-2180. PMID:26729617 doi:10.1111/ajt.13701
3. Geramita EM, DeVito Dabbs AJ, DiMartini AF, et al. Impact of a Mobile Health Intervention on Long-term Nonadherence After Lung Transplantation: Follow-up After a Randomized Controlled Trial. Transplantation. 2020;104(3):640-651. PMID:31335759 doi:10.1097/TP.0000000000002872
4. Gomis-Pastor M, Mirabet Perez S, Roig Minguell E, et al. Mobile Health to Improve Adherence and Patient Experience in Heart Transplantation Recipients: The mHeart Trial. Healthcare (Basel). 2021;9(4):463. PMID:33919899 doi:10.3390/healthcare9040463
5. Gomis-Pastor M, Mirabet Perez S, De Dios Lopez A, et al. Does an eHealth Intervention Reduce Complications and Healthcare Resources? A mHeart Single-Center Randomized-Controlled Trial. J Cardiovasc Dev Dis. 2023;10(2):77. PMID:36826572 doi:10.3390/jcdd10020077
6. Gonzales HM, Fleming JN, Gebregziabher M, et al. Pharmacist-Led Mobile Health Intervention and Transplant Medication Safety: A Randomized Controlled Clinical Trial. Clin J Am Soc Nephrol. 2021;16(5):776-784. PMID:33931415 doi:10.2215/CJN.15911020
7. Fleming JN, Gebregziabher M, Posadas A, et al. Impact of a pharmacist-led, mHealth-based intervention on tacrolimus trough variability in kidney transplant recipients: A report from the TRANSAFE Rx randomized controlled trial. Am J Health Syst Pharm. 2021;78(14):1287-1293. PMID:33821958 doi:10.1093/ajhp/zxab157
8. Han A, Min SI, Ahn S, et al. Mobile medication manager application to improve adherence with immunosuppressive therapy in renal transplant recipients: A randomized controlled trial. PLoS One. 2019;14(11):e0224595. PMID:31689320 doi:10.1371/journal.pone.0224595
9. Henriksson J, Tydén G, Höijer J, Wadström J. A Prospective Randomized Trial on the Effect of Using an Electronic Monitoring Drug Dispensing Device to Improve Adherence and Compliance. Transplantation. 2016;100(1):203-209. PMID:26588006 doi:10.1097/TP.0000000000000971
10. Hume E, Muse H, Wallace K, et al. Feasibility and acceptability of a physical activity behavioural modification tele-coaching intervention in lung transplant recipients. Chron Respir Dis. 2022;19:14799731221116588. PMID:36306548 doi:10.1177/14799731221116588
11. Lee TC, Kaiser TE, Alloway R, et al. Telemedicine Based Remote Home Monitoring After Liver Transplantation: Results of a Randomized Prospective Trial. Ann Surg. 2019;270(3):564-572. PMID:31356267 doi:10.1097/SLA.0000000000003425
12. McGillicuddy JW, Gregoski MJ, Weiland AK, et al. Mobile Health Medication Adherence and Blood Pressure Control in Renal Transplant Recipients: A Proof-of-Concept Randomized Controlled Trial. JMIR Res Protoc. 2013;2(2):e32. PMID:24004517 doi:10.2196/resprot.2633
13. McGillicuddy JW, Chandler JL, Sox LR, et al. Exploratory Analysis of the Impact of an mHealth Medication Adherence Intervention on Tacrolimus Trough Concentration Variability: Post Hoc Results of a Randomized Controlled Trial. Ann Pharmacother. 2020;54(12):1185-1193. PMID:32506922 doi:10.1177/1060028020931806
14. Sengpiel J, Fuehner T, Kugler C, et al. Use of telehealth technology for home spirometry after lung transplantation: a randomized controlled trial. Prog Transplant. 2010;20(4):310-317. PMID:21265282 doi:10.1177/152692481002000402
15. Tian M, Wang B, Xue Z, et al. Telemedicine for Follow-up Management of Patients After Liver Transplantation: Cohort Study. JMIR Med Inform. 2021;9(5):e27175. PMID:33999008 doi:10.2196/27175
16. Schenkel FA, Barr ML, McCloskey CC, et al. Use of a Bluetooth tablet-based technology to improve outcomes in lung transplantation: A pilot study. Am J Transplant. 2020;20(12):3649-3657. PMID:32558226 doi:10.1111/ajt.16154
17. Tian B, Lu H, Zhang J, et al. Application of Telemedicine Robot in Follow-up After Liver Transplantation From Donation After Cardiac Death. Organ Transplantation. 2019;10(1):79-83. doi: 10.3969/j.issn.1674-7445.2019.01.012
18. Serper M, Barankay I, Chadha S, et al. A randomized, controlled, behavioral intervention to promote walking after abdominal organ transplantation: results from the LIFT study. Transpl Int. 2020;33(6):632-643. PMID:31925833 doi:10.1111/tri.13570
