# Supplementary material for: Effectiveness of mHealth Interventions to Improve Follow-Up and Management Among Solid Organ Transplant Recipients: Systematic Review and Meta-Analysis
Source: JMIR Mhealth Uhealth. 2025 Dec 17;13:e69795. doi: 10.2196/69795 (PMC12756658; doi:10.2196/69795)
Supplement: Multimedia Appendix 5 [file mhealth_v13i1e69795_app5.docx]

**Multimedia Appendix 5. Risk of Bias Assessment**

1. Each risk of bias domain for each RCTs.


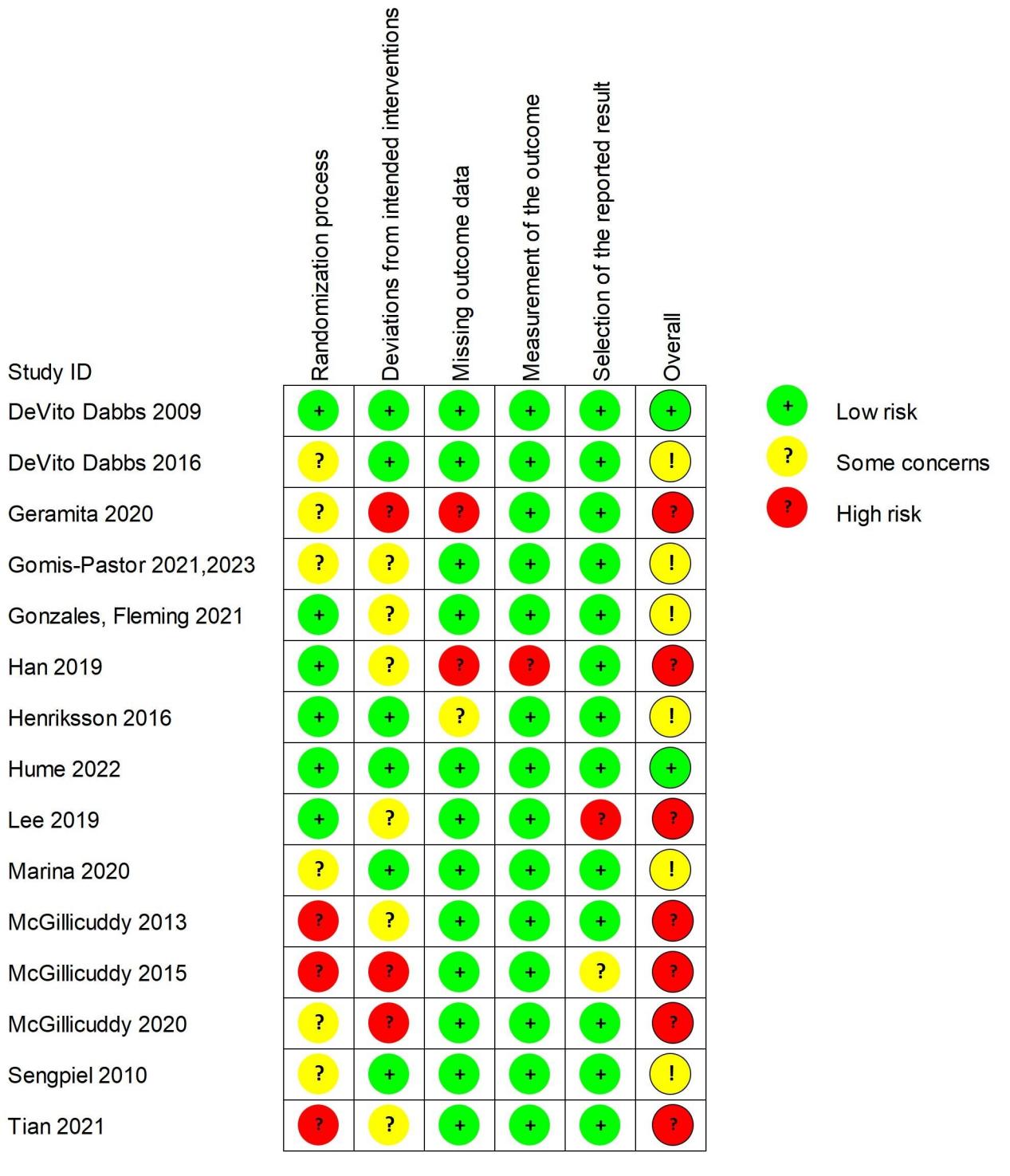


1. Each risk of bias domain presented as a percentage across RCTs.


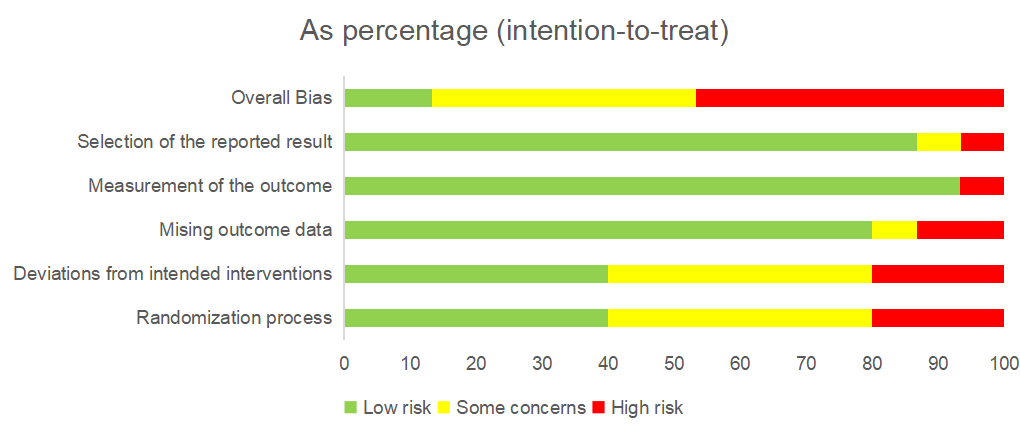


1. Each risk of bias domain for each NRSIs.


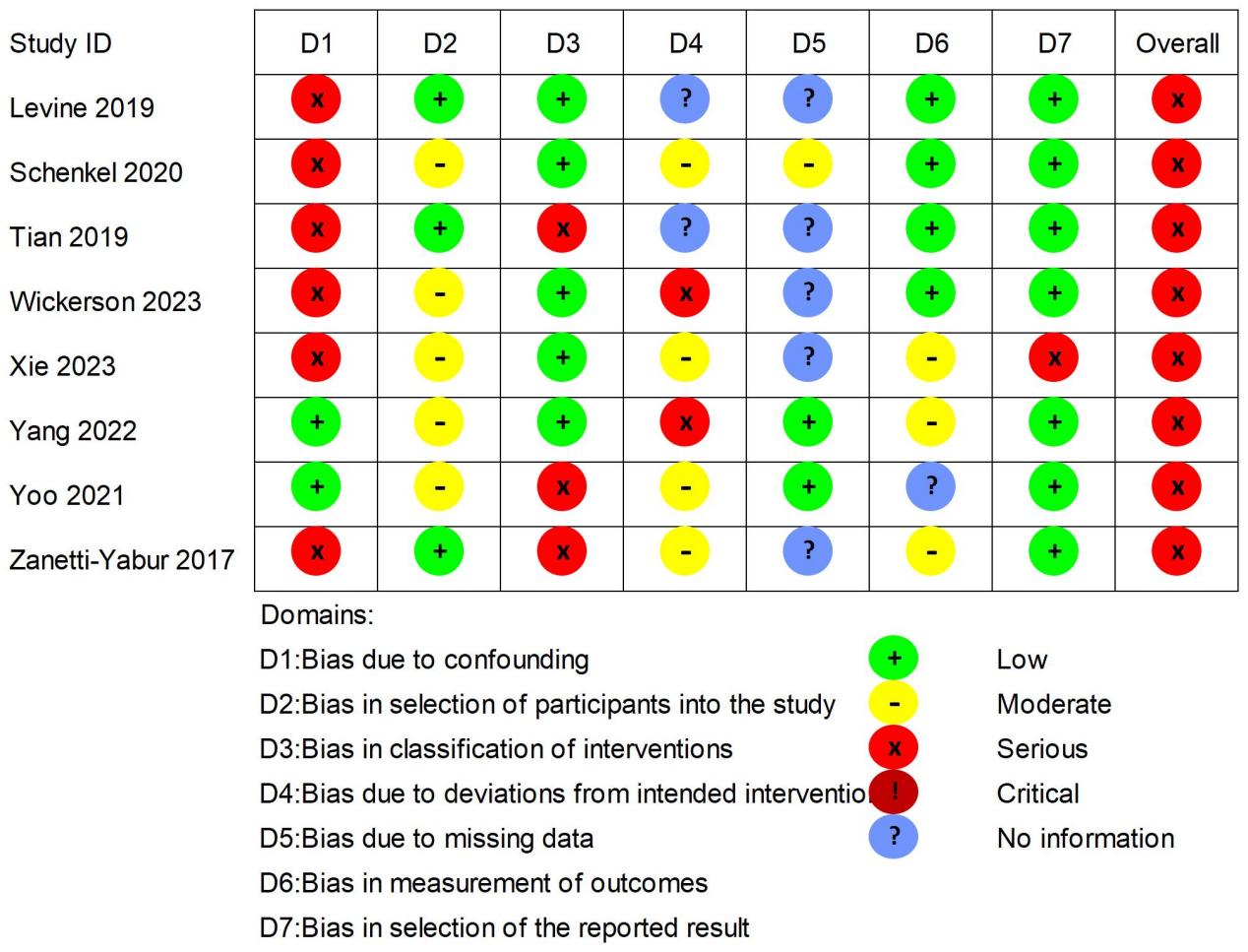


### **References**

1. DeVito Dabbs A, Dew MA, Myers B, et al. Evaluation of a hand-held, computer-based intervention to promote early self-care behaviors after lung transplant. Clin Transplant. 2009;23(4):537-545. PMID:19473201 doi:10.1111/j.1399-0012.2009.00992.x
2. Xie X, Wang X, Li A, et al. A Study of the Effectiveness of Mobile Health Application in A Self-management Intervention for Kidney Transplant Patients. Iran J Kidney Dis. 2023;17(5):263-270. doi: 10.52547/ijkd.7693
3. Yoo HJ, Suh EE. Effects of a smartphone-based self-care health diary for heart transplant recipients: A mixed methods study. Appl Nurs Res. 2021;58:151408. PMID:33745556 doi:10.1016/j.apnr.2021.151408
4. DeVito Dabbs A, Song MK, Myers BA, et al. A Randomized Controlled Trial of a Mobile Health Intervention to Promote Self-Management After Lung Transplantation. Am J Transplant. 2016;16(7):2172-2180. PMID:26729617 doi:10.1111/ajt.13701
5. Geramita EM, DeVito Dabbs AJ, DiMartini AF, et al. Impact of a Mobile Health Intervention on Long-term Nonadherence After Lung Transplantation: Follow-up After a Randomized Controlled Trial. Transplantation. 2020;104(3):640-651. PMID:31335759 doi:10.1097/TP.0000000000002872
6. Gomis-Pastor M, Mirabet Perez S, Roig Minguell E, et al. Mobile Health to Improve Adherence and Patient Experience in Heart Transplantation Recipients: The mHeart Trial. Healthcare (Basel). 2021;9(4):463. PMID:33919899 doi:10.3390/healthcare9040463
7. Gomis-Pastor M, Mirabet Perez S, De Dios Lopez A, et al. Does an eHealth Intervention Reduce Complications and Healthcare Resources? A mHeart Single-Center Randomized-Controlled Trial. J Cardiovasc Dev Dis. 2023;10(2):77. PMID:36826572 doi:10.3390/jcdd10020077
8. Gonzales HM, Fleming JN, Gebregziabher M, et al. Pharmacist-Led Mobile Health Intervention and Transplant Medication Safety: A Randomized Controlled Clinical Trial. Clin J Am Soc Nephrol. 2021;16(5):776-784. PMID:33931415 doi:10.2215/CJN.15911020
9. Fleming JN, Gebregziabher M, Posadas A, et al. Impact of a pharmacist-led, mHealth-based intervention on tacrolimus trough variability in kidney transplant recipients: A report from the TRANSAFE Rx randomized controlled trial. Am J Health Syst Pharm. 2021;78(14):1287-1293. PMID:33821958 doi:10.1093/ajhp/zxab157
10. Han A, Min SI, Ahn S, et al. Mobile medication manager application to improve adherence with immunosuppressive therapy in renal transplant recipients: A randomized controlled trial. PLoS One. 2019;14(11):e0224595. PMID:31689320 doi:10.1371/journal.pone.0224595
11. Henriksson J, Tydén G, Höijer J, Wadström J. A Prospective Randomized Trial on the Effect of Using an Electronic Monitoring Drug Dispensing Device to Improve Adherence and Compliance. Transplantation. 2016;100(1):203-209. PMID:26588006 doi:10.1097/TP.0000000000000971
12. Hume E, Muse H, Wallace K, et al. Feasibility and acceptability of a physical activity behavioural modification tele-coaching intervention in lung transplant recipients. Chron Respir Dis. 2022;19:14799731221116588. PMID:36306548 doi:10.1177/14799731221116588
13. Lee TC, Kaiser TE, Alloway R, et al. Telemedicine Based Remote Home Monitoring After Liver Transplantation: Results of a Randomized Prospective Trial. Ann Surg. 2019;270(3):564-572. PMID:31356267 doi:10.1097/SLA.0000000000003425
14. McGillicuddy JW, Gregoski MJ, Weiland AK, et al. Mobile Health Medication Adherence and Blood Pressure Control in Renal Transplant Recipients: A Proof-of-Concept Randomized Controlled Trial. JMIR Res Protoc. 2013;2(2):e32. PMID:24004517 doi:10.2196/resprot.2633
15. McGillicuddy JW, Taber DJ, Mueller M, et al. Sustainability of improvements in medication adherence through a mobile health intervention. Prog Transplant. 2015;25(3):217-223. PMID:26308780 doi:10.7182/pit2015975
16. McGillicuddy JW, Chandler JL, Sox LR, et al. Exploratory Analysis of the Impact of an mHealth Medication Adherence Intervention on Tacrolimus Trough Concentration Variability: Post Hoc Results of a Randomized Controlled Trial. Ann Pharmacother. 2020;54(12):1185-1193. PMID:32506922 doi:10.1177/1060028020931806
17. Sengpiel J, Fuehner T, Kugler C, et al. Use of telehealth technology for home spirometry after lung transplantation: a randomized controlled trial. Prog Transplant. 2010;20(4):310-317. PMID:21265282 doi:10.1177/152692481002000402
18. Tian M, Wang B, Xue Z, et al. Telemedicine for Follow-up Management of Patients After Liver Transplantation: Cohort Study. JMIR Med Inform. 2021;9(5):e27175. PMID:33999008 doi:10.2196/27175
19. Levine D, Torabi J, Choinski K, et al. Transplant surgery enters a new era: Increasing immunosuppressive medication adherence through mobile apps and smart watches. Am J Surg. 2019;218(1):18-20. PMID:30799019 doi:10.1016/j.amjsurg.2019.02.018
20. Schenkel FA, Barr ML, McCloskey CC, et al. Use of a Bluetooth tablet-based technology to improve outcomes in lung transplantation: A pilot study. Am J Transplant. 2020;20(12):3649-3657. PMID:32558226 doi:10.1111/ajt.16154
21. Tian B, Lu H, Zhang J, et al. Application of Telemedicine Robot in Follow-up After Liver Transplantation From Donation After Cardiac Death. Organ Transplantation. 2019;10(1):79-83. doi: 10.3969/j.issn.1674-7445.2019.01.012
22. Wickerson L, Rozenberg D, Singer LG, et al. Early Change in Lower Limb Strength and Function in Lung Transplant Patients After Center-Based and Telerehabilitation. J Cardiopulm Rehabil Prev. 2023;43(1):55-60. PMID:35961370 doi:10.1097/HCR.0000000000000728
23. Zanetti-Yabur A, Rizzo A, Hayde N, et al. Exploring the usage of a mobile phone application in transplanted patients to encourage medication compliance and education. Am J Surg. 2017;214(4):743-747. PMID:28256241 doi:10.1016/j.amjsurg.2017.01.026
24. Serper M, Barankay I, Chadha S, et al. A randomized, controlled, behavioral intervention to promote walking after abdominal organ transplantation: results from the LIFT study. Transpl Int. 2020;33(6):632-643. PMID:31925833 doi:10.1111/tri.13570
25. Yang JD, Song J, Zhu Q, Ye Q, Wu CZ. Management of "Internet plus" procedural follow-up program in renal transplant recipients. Chin J Gen Pract. 2022;20(7):1178-1181. doi:10.16766/j.cnki.issn.1674-4152.002552
